# MEN1 Deficiency‐Driven Activation of the β‐Catenin‐MGMT Axis Promotes Pancreatic Neuroendocrine Tumor Growth and Confers Temozolomide Resistance

**DOI:** 10.1002/advs.202308417

**Published:** 2024-07-23

**Authors:** Junfeng Xu, Xin Lou, Fei Wang, Wuhu Zhang, Xiaowu Xu, Zeng Ye, Qifeng Zhuo, Yan Wang, Desheng Jing, Guixiong Fan, Xuemin Chen, Yue Zhang, Chenjie Zhou, Jie Chen, Yi Qin, Xianjun Yu, Shunrong Ji

**Affiliations:** ^1^ Center for Neuroendocrine Tumors Fudan University Shanghai Cancer Center Shanghai 200032 China; ^2^ Department of Pancreatic Surgery Fudan University Shanghai Cancer Center Shanghai 200032 China; ^3^ Department of Oncology Shanghai Medical College Fudan University Shanghai 200032 China; ^4^ Shanghai Pancreatic Cancer Institute Shanghai 200032 China; ^5^ Pancreatic Cancer Institute Fudan University Shanghai 200032 China; ^6^ The First People's Hospital of Changzhou The Third Affiliated Hospital of Soochow University Changzhou 213004 China

**Keywords:** MEN1, MGMT, Pancreatic neuroendocrine tumors, temozolomide

## Abstract

O6‐methylguanine DNA methyltransferase (MGMT) removes alkyl adducts from the guanine O6 position (O^6^‐MG) and repairs DNA damage. High MGMT expression results in poor response to temozolomide (TMZ). However, the biological importance of MGMT and the mechanism underlying its high expression in pancreatic neuroendocrine tumors (PanNETs) remain elusive. Here, it is found that MGMT expression is highly elevated in PanNET tissues compared with paired normal tissues and negatively associated with progression‐free survival (PFS) time in patients with PanNETs. Knocking out MGMT inhibits cancer cell growth in vitro and in vivo. Ectopic MEN1 expression suppresses MGMT transcription in a manner that depends on β‐Catenin nuclear export and degradation. The Leucine 267 residue of MEN1 is crucial for regulating β‐Catenin‐MGMT axis activation and chemosensitivity to TMZ. Interference with β‐Catenin re‐sensitizes tumor cells to TMZ and significantly reduces the cytotoxic effects of high‐dose TMZ treatment, and MGMT overexpression counteracts the effects of β‐Catenin deficiency. This study reveals the biological importance of MGMT and a new mechanism by which MEN1 deficiency regulates its expression, thus providing a potential combinational strategy for treating patients with TMZ‐resistant PanNETs.

## Graphical abstract

Temozolomide (TMZ) is a first‐in‐class clinical chemotherapeutic drug for the treatment of advanced PanNETs via inducing DNA damage, while MGMT repairs DNA damage and disrupts the TMZ response. This study reveals that MEN1 deficiency induces activation of the β‐Catenin‐MGMT axis, and the restoration of MEN1 or the suppression of β‐Catenin re‐sensitizes PanNETs to TMZ.

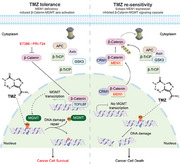

## 1 Introduction

Multiple endocrine neoplasia type 1 (MEN1) syndrome, a disease susceptibility to tumors driven by MEN1 deficiency mutation, is characterized by sporadic occurrence of neuroendocrine tumors in human organs.^[^ ^1^ ^]^ Pancreatic neuroendocrine tumors (PanNETs), which are rare tumors, have a relatively low incidence and account for a small proportion of pancreatic tumors. PanNETs are highly heterogeneous, and they are classified into nonfunctional PanNETs and functional PanNETs based on the bioactive hormones that they secrete.^[^ ^2^ ^]^ In addition, the 2017 World Health Organization (WHO) classification system stratifies PanNETs into well‐differentiated NETs and poorly differentiated pancreatic neuroendocrine carcinomas according to their Ki67 index, mitotic index, and histopathological differentiation. Although mutations in MEN1, DAXX (death domain associated protein), ATRX (alpha‐thalassemia‐X‐linked intellectual disability syndrome), and Tumor Protein 53 (TP53) are more likely to be responsible for promoting PanNET evolution and advancement,^[^ ^3^ ^]^ the heterogeneity and mechanism underlying tumor malignancy are still unclear.

MEN1 is the most commonly mutated gene and a typical tumor suppressor in PanNETs. According to whole‐genome sequencing studies, more than 30% of primary PanNETs harbor somatic mutations.^[^ ^4^, ^5^ ^]^ Our recent study using whole exome sequencing (WES) showed a consistent result that MEN1 had the highest frequency of mutations,^[^ ^6^ ^]^ and these mutations were associated with the loss of its function, suggesting that the MEN1 gene plays a leading role in driving PanNET tumorigenesis and development. The MEN1‐encoded protein menin was shown to shuttle between the cytoplasm and the nucleus.^[^ ^7^, ^8^, ^9^ ^]^ In the nucleus, menin likely functions as an essential scaffold protein that interacts with chromatin‐modified proteins or transcription factors in response to stimuli in the extracellular environment (e.g., DNA damaging agents and growth factors) and modulates multiple signaling pathways, such as the Nuclear Factor Kappa B (NF‐κB), TGF‐β (Transforming growth factor beta), Hedgehog and DNA damage repair signaling pathways.^[^ ^10^, ^11^, ^12^, ^13^ ^]^ Our previous study showed that cytoplasmic MEN1 can promote monounsaturated fatty acid metabolism and attenuate mechanistic target of rapamycin kinase (mTOR) activation.^[^ ^6^ ^]^ Moreover, MEN1 overexpression was further shown to induce PanNET cell ferroptosis and suppress cancer cell growth via the inhibition of stearoyl‐CoA desaturase 1 (SCD1).^[^ ^7^ ^]^ Importantly, MEN1 was reported to directly bind to β‐Catenin and transport β‐Catenin out of the nucleus via the nuclear pore complex‐dependent nuclear export receptor chromosome region maintenance 1 (CRM1), thus reducing β‐Catenin nuclear localization and its transcriptional activity.^[^ ^8^ ^]^ Therefore, these results suggest that MEN1 plays a key role both in the nucleus and in the cytoplasm. However, the important functions of MEN1 in PanNETs have not yet been fully elucidated.

Temozolomide (TMZ) is a small lipophilic alkylating agent that reacts with DNA bases to form methyl adducts, such as O6‐methylguanine (O^6^‐MG), which induce DNA damage, cell cycle arrest, and cell death.^[^ ^14^, ^15^ ^]^ TMZ is a first‐line clinical chemotherapeutic drug that has been widely used in patients with advanced PanNETs. However, recent clinical trials demonstrated that compared with MGMT deficiency, a high level of MGMT was associated with a worse response to TMZ treatment,^[^ ^16^ ^]^ suggesting that MGMT might be a potent target of clinical interventions that aim to restore the sensitivity of PanNETs to TMZ. MGMT (O6 ‐ methylguanine DNA methyltransferase)  is an evolutionarily highly conserved DNA repair enzyme that has few mutations in tumors. MGMT is characterized by its dealkylating effect, which involves transferring methyl groups from O6‐methylguanine to its cysteine residues to repair damaged DNA.^[^ ^17^ ^]^ MGMT functions as a double‐edged sword; low levels of MGMT lead to DNA damage repair and maintain genomic integrity, whereas high levels of MGMT antagonize chemotherapy. In addition to removing alkyl adducts from DNA lesions, MGMT is highly expressed in pancreatic cancer,^[^ ^18^ ^]^ and the suppression of MGMT activity by the pharmaceutical compound O6‐benzylguanine (O6‐BG) decreases cancer cell proliferation and induces cell apoptosis;^[^ ^19^ ^]^ these results indicate that MGMT can also play a biologically important role in tumor development. Recent studies have demonstrated that MGMT is regulated at multiple levels via distinct molecular mechanisms in brain cancer and colon cancer;^[^ ^20^, ^21^ ^]^ the mechanisms by which it is regulated include genomic promoter methylation with epigenetic silencing,^[^ ^22^, ^23^, ^24^ ^]^ transcriptional control by transcription factors and cofactors,^[^ ^25^, ^26^, ^27^ ^]^ posttranscriptional repression by miRNAs,^[^ ^28^, ^29^ ^]^ and posttranslational modification by E3 ligases.^[^ ^30^ ^]^ Specifically, the canonical Wnt/β‐Catenin cascade was shown to promote MGMT expression at the transcription level.^[^ ^31^, ^32^ ^]^ Inhibition of Wnt signaling by selective Wnt inhibitors or RNA interference decreases MGMT transcription and enhances the effects of alkylating agents by restoring tumor chemosensitivity in mouse models of human brain cancer.^[^ ^31^ ^]^ However, no studies on the role of MGMT and the regulation of its expression in PanNETs have been reported. A better understanding of the underlying regulatory mechanisms that are responsible for high MGMT expression is needed to better guide decisions related to the treatment of patients with PanNETs.

In this study, we reported for the first time the biological importance of MGMT and a new mechanism by which MGMT expression is regulated by MEN1, that is, the loss of MEN1controlled MGMT transcription and tumor chemosensitivity to TMZ via the activation of β‐Catenin. High expression of MGMT was observed in patients with PanNETs and was associated with poor outcomes. Depletion of MGMT led to cancer cell cycle arrest and cell apoptosis. Ectopic MEN1 expression downregulated MGMT expression and re‐sensitized cancer cells to TMZ by suppressing the nuclear accumulation and transcriptional activity of β‐Catenin. The Leucine 267 residue of MEN1 was needed to control β‐Catenin‐MGMT axis activation and the response to TMZ. Pancreas‐specific MEN1‐knockout (KO) mice and clinical patient specimens were used to further confirm the inverse correlation between MEN1 and MGMT. Our study revealed a previously unknown regulatory function of MEN1 in PanNETs and provides a potential new combination strategy for treating patients with TMZ‐resistant PanNETs.

## 2 Results

### 2.1 High MGMT Expression was Associated with a Poor Prognosis in Patients with PanNETs and Promoted Tumor Growth In Vivo

**Figure 1:**
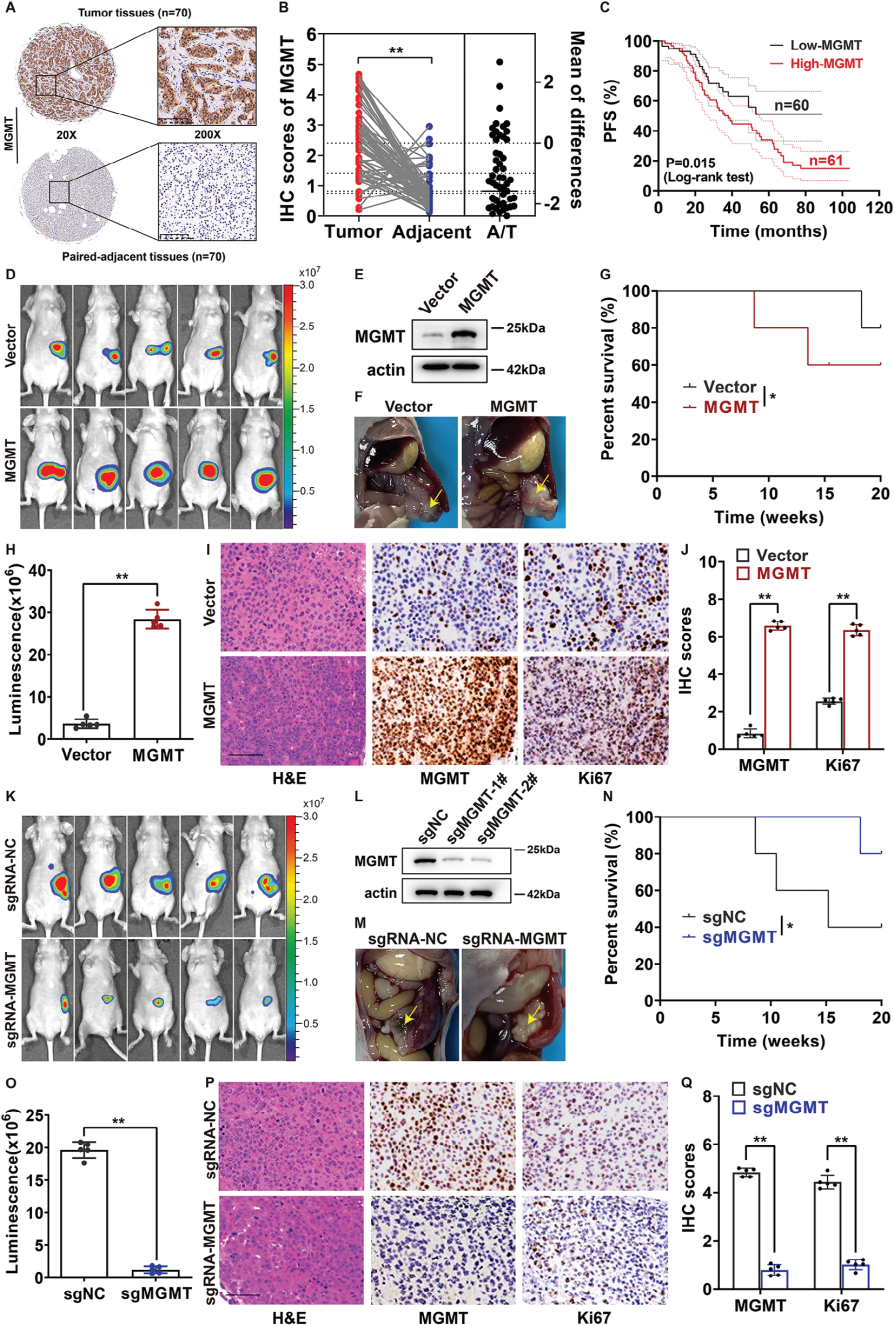
High level of MGMT expression was associated with a poor prognosis in patients with PanNETs and promoted tumor growth in vivo. A) Immunohistochemistry (IHC) staining of PanNET tissue microarrays using MGMT‐specific antibody. (*n* = 70). Scale bar for ×200 images: 100µm. B) The level of MGMT expression was significantly higher in PanNET tissues than in paired adjacent normal tissues in IHC staining of tissue arrays. (**P < 0.01 as determined by t‐test, *n* = 70). C) Kaplan‐Meier analysis of PFS rate of patients with PanNETs according to the level of MGMT expression, respectively. (*n* = 121, *P *= 0.015 as determined by log‐rank test). D–G 1x106 MGMT‐overexpressing stable Bon‐1 cells were injected into orthotopic pancreas of nude mice (*n* = 5); H–K 1x106 MGMT‐knocking out stable QGP‐1 cells were injected into orthotopic pancreas of nude mice (*n* = 5). D,K) Fluorescence of lesions generated in nude mice orthotopic pancreas individually injected with stably transfected PanNET cells or control cells was examined, *n* = 5. E,L) The protein levels of MGMT in stably transfected Bon‐1 or QGP‐1 cells were examined by Western blot. F,M) Gross anatomy of the pancreas in mouse models at the age of 3 weeks. G,N) Survival of mice in the orthotopic pancreas‐seeded mouse models. Bon‐1‐MGMT and control cells, *n* = 5, *P < 0.05; QGP‐1‐sgRNA‐MGMT and control cells, *n* = 5, *P < 0.05, log‐rank test. H,O) Statistical analysis of luciferase intensities in mouse models at the age of 3 weeks. Bon‐1‐MGMT and control cells, *n* = 5, **P < 0.01; QGP‐1‐sgRNA‐MGMT and control cells, *n* = 5, **P < 0.01, log‐rank test. I,P) Immunohistochemistry (IHC) staining of the lesions generated in mouse models using MGMT or Ki67 specific antibodies. (*n* = 5). Scale bar, 200µm. J,Q) Statistical analysis of IHC scores in mouse models. For the expression of Ki67 and MGMT, Bon‐1‐MGMT vs control cells, *n* = 5, **P < 0.01; For the expression of Ki67 and MGMT, QGP‐1‐sgRNA‐MGMT and control cells, *n* = 5, **P < 0.01. These data were representative of three independent experiments. Data represented means, and error bars were standard deviations. Two‐sided t‐test.

To assess the role of MGMT in PanNETs, we first examined its expression levels by immunohistochemistry (IHC) staining of tissue microarrays (TMAs) from 121 PanNET samples. These samples included 70 paired primary tumor tissue samples and adjacent normal tissue samples. We observed a higher level of MGMT expression in tumor tissues than paired normal tissues (*p* < 0.05) (**Figure** **1**A,B). The distribution of clinicopathological characteristics of those patients and the expression level of MGMT stratified by patient characteristics are shown in Tables S1 and S2 (Supporting Information). Half of the patients were women, and half of the patients were aged 55 years and older. More than one‐third of patients had tumors larger than 4 cm. Most patients were diagnosed with tumors in the body and tail of the pancreas, 35.5% of the patients were diagnosed with tumors in the head of the pancreas, and only 5.8% of the patients were diagnosed with tumors in other parts of the pancreas. A total of 17.4% of patients had tumors at the T1 stage, 52.1% of patients had tumors at the T2 stage, 22.3% of patients had tumors at the T3 stage and 8.2% of patients had tumors at the T4 stage according to the European Neuroendocrine Tumors Society (ENETS) system and the 8th edition of the American Joint Committee on Cancer (AJCC) staging system for PanNETs. Two‐thirds of patients were diagnosed with no regional lymph node metastasis, and the rest had at least one lymph node metastasis. Most of the patients had tumors in the M0 stage without metastatic lesions, and 14% of patients had tumors in the M1 stage with metastatic lesions. G1 cases accounted for 30.6% of all cases, G2 cases accounted for 61.2% of all cases and G3 cases accounted for 8.2% of all cases. No significant differences were observed between the expression levels of MGMT and the selected clinicopathological characteristics, including patient staging, tumor location, tumor grade, tumor size, and lymph node metastasis (Table S2, Supporting Information). We next assessed the association between MGMT expression and the prognosis of patients with PanNETs. As shown in Table S3 (Supporting Information), patients with higher levels of MGMT had significantly poorer outcomes, with a hazard ratio (HR) of 1.88, after adjusting for age, sex, tumor size, tumor location, and T/N/M stage. Kaplan–Meier survival analysis revealed that high levels of MGMT were negatively associated with PFS times of patients (log‐rank *P* = 0.015) (Figure 1C). Due to the small number of databases with data on the rare tumor, the mRNA levels of MGMT in human PanNET tissues and adjacent tissues were not significantly different according to the Gene Expression Omnibus (GEO) dataset (GSE73338) (Figure S1A, Supporting Information), whereas analysis of mouse GEO data (GSE248606) indicated that the expressions of MGMT were higher in PanNET tissues than in adjacent tissues (Figure S1B, Supporting Information).

These findings prompted us to explore the role of MGMT in PanNET progression. We constructed stable MGMT‐overexpressing (Figure 1E) and MGMT‐silenced (Figure 1L) cell lines using a lentivirus system, and the effect of MGMT on tumor growth was evaluated in an orthotopic pancreatic neuroendocrine tumor model (Figure 1D,K). Three weeks after injection, compared with the negative control group, the overexpression of MGMT notably promoted orthotopic pancreas‐seeded tumor growth (*n* = 5, *p* < 0.05), according to the gross anatomy of the mouse pancreas (Figure 1F) and the fluorescence intensity of the lesion (Figure 1H), which was associated with poorer survival (Figure 1G). Importantly, immunohistochemistry (IHC) of mouse tumors revealed that overexpression of MGMT markedly increased the expression of Ki67 compared with the vector control treatment (Figure 1I,J). In contrast, smaller tumors were observed in the MGMT‐knockdown group than in the negative control group (Figure 1M,O), and mice in the QGP‐1/sgRNA‐MGMT group had longer survivals than those in the negative control group (Figure 1N). Compared with the negative control cells, QGP‐1/sgRNA‐MGMT cells exhibited reduced expression of Ki67 in the mouse model (Figure 1P,Q). Taken together, these data indicate that MGMT plays an oncogenic role in PanNETs.

### 2.2 MGMT Loss Inhibited the Malignant Biological Behaviors of PanNETs In Vitro

**Figure 2:**
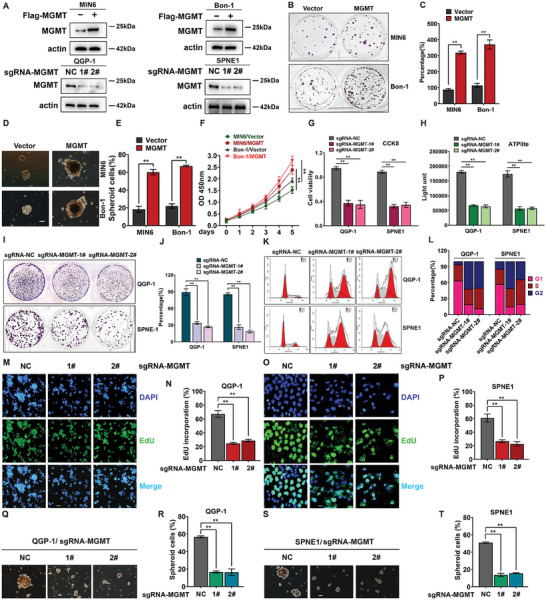
MGMT loss inhibited the tumor malignant biological behaviors of PanNETs in vitro. A) The protein levels of MGMT in stably transfected MIN6, Bon‐1, QGP‐1, or SPNE1 cells were examined by Western blot. B,C) Overexpression of MGMT promoted colony formation in MIN6 and Bon‐1 cells. 200 MGMT‐or control‐overexpressing stable MIN6 or Bon‐1 cells were seeded individually into 6‐well plates for 10 days, followed by crystal violet staining and colony counting (mean ± SD, **P < 0.01, *n* = 3). D,E) Overexpression of MGMT promoted cell spheroid‐forming ability in MIN6 and Bon‐1 cells. 1X104 MGMT‐or control‐overexpressing stable MIN6 or Bon‐1 cells were seeded individually into 6‐well plates with ultra‐low attachment surface (#3471, Corning, Kennebunk, ME) for 4 weeks, followed by the micrograph and spheroid counting (mean ± SD, **P < 0.01, *n* = 3). Scale bar, 100µm. F) Overexpression of MGMT facilitated cancer cell proliferation. 2,500 MGMT‐ or negative control‐ overexpressing stable MIN6 and Bon‐1 cells were seeded individually in 96‐well plates in triplicates and then subjected to CCK8 assay for indicated time points (mean ± SD, **P < 0.01, *n* = 3). G,H) MGMT knockout inhibited cancer cell proliferation. 2,500 MGMT‐ or negative control‐ knocking out stable QGP‐1 or SPNE1 cells were seeded individually in 96‐well plates in triplicates and then subjected for CCK8 and ATPlite assay (mean ± SD, **P < 0.01, *n* = 3). I,J) MGMT knockout inhibited cancer cell colony formation. 300 MGMT‐ or negative control‐ knocking out stable QGP‐1 or SPNE1 cells were seeded individually in 6‐well plates in triplicates for 10 days and then subjected to colony formation assay (mean ± SD, **P < 0.01, *n* = 3). K,L) MGMT knockout induced G2‐M cell‐cycle defects. 1x106 MGMT‐ or negative control‐ knocking out stable QGP‐1 or SPNE1 cells were subjected to PI staining and FACS analysis for cell‐cycle profile. (mean ± SD, *P* < 0.05, *n* = 3). M,N) EdU incorporation assays showed the inhibitory effect of MGMT knockout on QGP‐1 cell proliferation. MGMT‐ or negative control‐ knocking out stable QGP‐1 cells were subjected to EdU incorporation with Alexa Fluor 488 and imaging under a fluorescence imaging system. (mean ± SD, **P < 0.01, *n* = 3). Scale bar, 100 µm. O,P) EdU incorporation assays showed the inhibitory effect of MGMT knockout on SPNE1 cell proliferation. MGMT‐ or negative control‐ knocking out stable SPNE1 cells were subjected to EdU incorporation with Alexa Fluor 488 and imaging under a fluorescence imaging system. (mean ± SD, **P < 0.01, *n* = 3). Scale bar, 100 µm. Q–T) MGMT knockout inhibited cell spheroid‐forming ability in QGP‐1 and SPNE1 cells. 1X104 MGMT‐or control‐knocking out stable QGP‐1 or SPNE1 cells were seeded individually into 6‐well plates with ultra‐low attachment surface (#3471, Corning, Kennebunk, ME) for 4 weeks, followed by the micrograph and spheroid counting (mean ± SD, **P < 0.01, *n* = 3). Scale bar, 100 µm. These data were representative of three independent experiments. Data represented means, and error bars were standard deviations. Two‐sided t‐test.

Although a high level of MGMT expression was observed, the biological function of MGMT in PanNETs remains unclear. To investigate the role of MGMT in PanNET progression, we examined the expression level of MGMT in various human and mouse pancreatic cancer cell lines (SPNE1, NIT‐1, QGP‐1, Beta‐TC6, MIN6, and Bon‐1). We found that the expression levels of MGMT were significantly greater in the SPNE1, NIT‐1, and QGP‐1 cell lines than in the MIN6 and Bon‐1 cell lines (Figure S2, Supporting Information). Therefore, we explored the tumor malignant phenotypes influenced by the up‐regulation of MGMT expression in relatively low‐MGMT cell lines (MIN6 and Bon‐1) and the downregulation of MGMT expression in relatively high‐MGMT cell lines (QGP‐1 and SPNE1). As shown in **Figure** **2**A, we constructed stable MGMT‐overexpressing cell lines (MIN6 and Bon‐1) and stable MGMT knockdown cell lines (SPNE1 and QGP‐1) using a lentivirus system. As shown in Figure 2B,C, overexpression of MGMT markedly promoted the proliferation of MIN6 and Bon‐1 cells, as determined via colony formation analysis. Consistently, enhanced cell spheroid‐forming ability (Figure 2D,E) and increased cell growth according to the Cell Counting Kit‐8 (CCK‐8) assay were observed in two stable MGMT‐expressing cells (Figure 2F). In contrast, Transwell assays showed that compared with vector control, the overexpression of MGMT did not affect cell migration (Figure S3A, Supporting Information).

We then evaluated the biological behavior effects of knocking out MGMT in QGP‐1 and SPNE1 cells by using the CRISPR‒Cas9 system. As shown in Figure 2G,H, compared with the negative control, MGMT KO notably inhibited cell proliferation, as observed in the CCK8 and ATPlite assays, compared with the negative control. Further cell colony formation analysis demonstrated that the clonogenic survival of the two PanNET cell lines was reduced by MGMT silencing (Figure 2I,J). In addition, fluorescence‐activated cell sorting (FACS) analysis revealed that MGMT KO could lead to cell cycle arrest in the G2 phase compared with the negative control (Figure 2K,L). Consistently, MGMT KO‐induced inhibition of PanNET cell proliferation was confirmed by an EdU incorporation assay (Figure 2M–P). Importantly, as shown in Figure 2Q–T, compared with negative control, the loss of MGMT impaired the spheroid‐forming abilities of the two PanNET cell lines. Conversely, the knockout of MGMT had no effect on cell migration compared with negative control (Figure S3B, Supporting Information). Overall, these results suggested that MGMT has a cancer‐promoting function and controls PanNET cell growth and that the loss of MGMT suppressed tumor development.

### 2.3 MGMT Loss Induced p21 Accumulation and the Apoptosis in PanNET Cells

**Figure 3:**
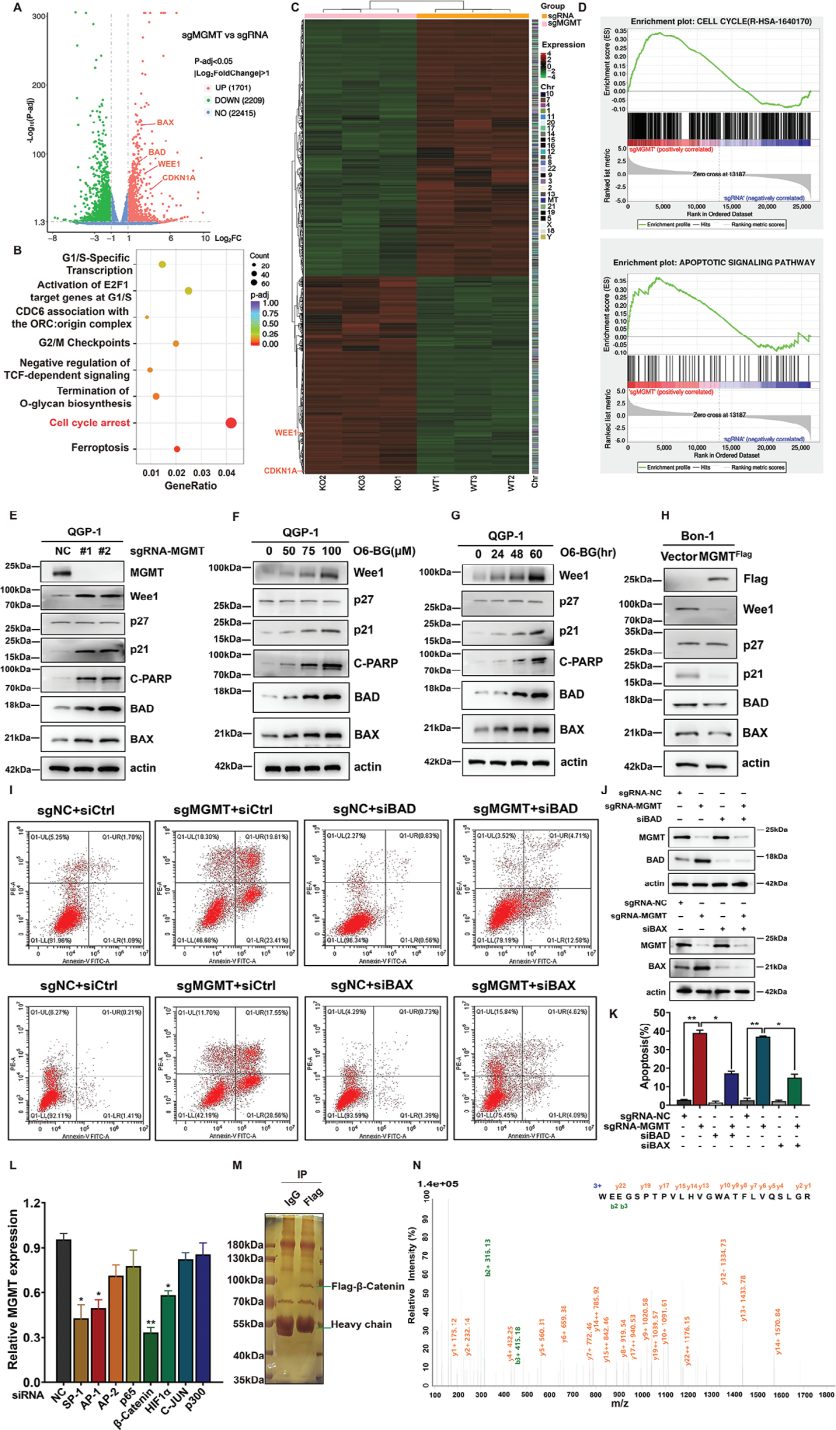
MGMT loss induced p21 accumulation and the apoptosis in PanNET cells. A–D) MGMT‐ or negative control‐ knocking out stable QGP‐1 cells were subjected to transcriptome sequencing. A) A volcano plot showed the significant up‐regulation of genes in MGMT‐knocking out stable QGP‐1 cells. The red dots (Right) indicated the significantly up‐regulated proteins and the green dots (Left) indicated the highly down‐regulated proteins (*P* < 0.05). B) Gene Ontology (GO) enrichment analysis showed cancer cell cycle arrest induced by MGMT‐knockout. C) Heatmap of differentially expressed proteins between the sgRNA‐negative control and sgRNA‐MGMT stable QGP‐1 cells. The red and green colors represented up‐ and down‐regulated proteins based on the ratio of the peak intensities, respectively. D) The significantly differentially expressed genes upon MGMT knockout were analyzed for their enrichment in gene datasets from RNAseq. The GSEA enrichment plots show enrichment scores on the y‐axis and genes ranked on the x‐axis. E) MGMT knockout led to the upregulation of Wee1, p21, cleaved‐PARP, BAD, and BAX. MGMT‐ or negative control‐ knocking out stable QGP‐1 cells were subjected to immunoblotting using antibodies against the indicated proteins with actin as a loading control. F) O6‐BG induced the accumulation of Wee1, p27, p21, C‐PARP, BAD, and BAX in a dose‐dependent manner. Forty‐eight hours after QGP‐1 cells treated with O6‐BG at increasing concentrations (50, 75, and 100 µm) vs DMSO, cells were subjected to immunoblotting using antibodies against the indicated proteins with actin as a loading control. G) O6‐BG induced the accumulation of Wee1, p27, p21, C‐PARP, BAD, and BAX in a time‐dependent manner. QGP‐1 cells were treated with 75 µm O6‐BG at increasing time points, and then subjected to immunoblotting using antibodies against the indicated proteins with actin as a loading control. H) Overexpression of MGMT led to the downregulation of Wee1, p21, BAD, and BAX. MGMT‐ or negative control‐ overexpressing stable Bon‐1 cells were subjected to immunoblotting using antibodies against the indicated proteins with actin as a loading control. I,K) BAD or BAX knockout partially rescued MGMT knockout‐induced cell apoptosis. MGMT‐ or negative control‐ knocking out stable QGP‐1 cells were individually transfected by siBAD, siBAX or siCtrl, and subjected to FACS analysis for cell apoptosis. (mean ± SD, **P < 0.01, *P < 0.05, *n* = 3). J) The protein levels of BAD, BAX, and MGMT in stably transfected QGP‐1 cells were examined by Western blot. L) Quantification of MGMT mRNA expression in the knockdown of reported different transcription factors. QGP‐1 cells were transfected individually by siControl, siSP‐1, siAP‐1, siAP‐2, sip65, siβ‐Catenin, siHIF1α, siC‐JUN, or sip300 for 72 h, and subjected to RT‐qPCR analysis for MGMT mRNA with actin as a control (mean ± SD, **P < 0.01, *P < 0.05, *n* = 3). M) Silver staining of SDS‐PAGE gels indicated β‐Catenin‐interacting proteins. Flag‐β‐Catenin expression stable QGP‐1 cells were lysed and immunoprecipitated with anti‐Flag Ab or rabbit IgG control Ab, and then subjected to SDS‐PAGE gel and silver staining. N) Representative tandem MS spectrum of the WEEGSPTPVLHVGWATFLVQSLGR peptide from MEN1 as determined by IP‐Mass Spec. These data were representative of three independent experiments. Data represented means, and error bars were standard deviations. Two‐sided t‐test.

To determine the molecular mechanism by which MGMT KO suppressed cell growth, a transcriptome sequencing (RNAseq) approach was used to compare the differentially expressed genes between stable MGMT‐KO QGP‐1 cells and the negative control. A total of 1701 genes had upregulated expression and 2209 genes had downregulated expression in stable sgRNA‐MGMT versus sgRNA‐negative control (NC) cells (**Figure** **3**A; Figure S4, Supporting Information). Among these genes, the expression of multiple genes associated with cell cycle arrest (e.g., Wee1; and p21) and apoptotic signaling pathways (e.g., BAX, BCL2 associated X, apoptosis regulator; and BAD, BCL2 associated agonist of cell death) were significantly upregulated (Figure 3A,C). Geneontology(GO) enrichment analysis revealed that the biological function of cell cycle arrest was largely activated in MGMT‐KO cells compared with negative control cells (Figure 3B). Gene set enrichment analysis (GSEA) also showed a consistent result, namely, that a set of genes associated with the cell cycle and cell apoptosis had notably upregulated expression in MGMT‐KO cells compared with negative control cells (Figure 3D).

To validate RNAseq results, we determined the levels of proteins associated with cell cycle arrest and cell apoptosis in stable MGMT‐KO QGP‐1 cells by western blot analysis. We found that the expression of p21, cleaved‐PARP, BAD, and BAX was markedly upregulated (Figure 3E), which was consistent with previous studies.^[^ ^19^ ^]^ In addition, O6‐BG, which is an MGMT‐inactivating small compound, increased p21, cleaved‐PARP, BAD, and BAX expression in a dose‐ and time‐dependent manner (Figure 3F,G). In contrast, overexpression of MGMT decreased the levels of p21, BAD, and BAX in Bon‐1 cells (Figure 3H). These data suggest that BAD and BAX play a causal role in the induction of apoptosis upon MGMT loss. To test this hypothesis, as the expression of BAD or BAX was downregulated via siRNA silencing in QGP‐1 cells, its effect on the induction of apoptosis upon MGMT loss was examined (Figure 3J). As shown in Figure 3I,K, knocking down BAD or BAX partially alleviated the cell apoptosis induced by MGMT knockout in QGP‐1 cells, indicating that the MGMT loss‐induced upregulation of BAD or BAX expression may play a critical role in determining cell fate.

Few mutations in MGMT have been detected in numerous whole‐genome sequencing studies,^[^ ^4^, ^5^, ^6^ ^]^ and whether the methylation level of the MGMT promoter accounts for its expression level is controversial;^[^ ^33^ ^]^ thus, we hypothesized that there is a particular unique signaling cascade in PanNETs that regulates the high levels of MGMT in tumors. Based on this hypothesis, we first screened multiple previously reported transcription factors and examined the mRNA expression of MGMT after downregulation or upregulation of its expression using RNA interference or overexpression approaches. As shown in Figure 3L, knockdown of SP‐1, AP‐1, β‐Catenin or hypoxia inducible factor 1 subunit alpha (HIF1α) individually decreased MGMT mRNA expression. In contrast, overexpression of β‐Catenin significantly increased the mRNA expression and transcription of MGMT, whereas overexpression of p53 reduced MGMT mRNA expression (Figure S5A,B, Supporting Information); these results indicated that β‐Catenin could play a leading role in the positive regulation of MGMT expression and that p53 may negatively regulate MGMT expression.

β‐Catenin is a common transcription factor that is activated in multiple tumors. The β‐Catenin‐MGMT functional axis has been ubiquitously reported,^[^ ^31^, ^32^ ^]^ but the factors that control the β‐Catenin‐MGMT signaling cascade in this rare tumor are still unknown. We next used immunoprecipitation coupled mass spectrometry (IP‐MS) and stable Flag‐β‐Catenin‐expressing QGP‐1 cells to identify a potentially important regulator (Figure 3M). In addition to the few known β‐Catenin binding proteins, such as beta‐transducin repeat containing E3 ubiquitin protein ligase (β‐TrCP), 3 specific peptide sequences of MEN1 were also identified via IP mass spectrometry (Figure 3N; Figure S6A,B, Supporting Information). Taken together, these data collectively showed that MGMT deficiency upregulated p21 expression and increased tumor cell apoptosis, and these data further suggested that MEN1 is involved in the β‐Catenin‐MGMT signaling cascade in PanNETs.

### 2.4 MEN1 Regulated MGMT Transcription and was Negatively Correlated with MGMT Levels in PanNETs

**Figure 4:**
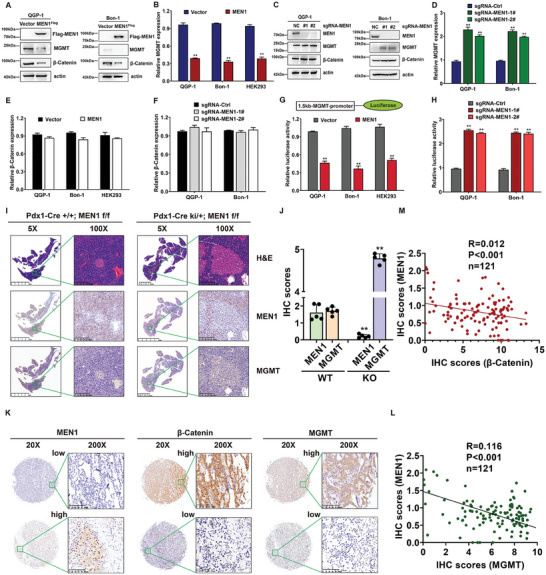
MEN1 regulated MGMT transcription and was negatively correlated with MGMT levels in PanNETs. A) Overexpression of MEN1 led to downregulation of the MGMT protein level in PanNET cells. MEN1‐ or negative control‐ overexpressing stable QGP‐1 and Bon‐1 cells were subjected to immunoblotting using antibodies against Flag, MGMT and β‐Catenin with actin as a loading control. B) Overexpression of MEN1 led to downregulation of the MGMT mRNA level. MEN1‐ or negative control‐ overexpressing stable QGP‐1, Bon‐1, and HEK293 cells were subjected to RT‐qPCR analysis for MGMT mRNA with actin as a control (mean ± SD, **P < 0.01, *n* = 3). C) Knockout of MEN1 led to upregulation of the MGMT protein level in PanNET cells. MEN1‐ or negative control‐ knocking out stable QGP‐1 and Bon‐1 cells were subjected to immunoblotting using antibodies against MEN1, MGMT, and β‐Catenin with actin as a loading control. D) Knockout of MEN1 led to upregulation of the MGMT mRNA level in PanNET cells. MEN1‐ or negative control‐ knocking out stable QGP‐1 and Bon‐1 cells were subjected to RT‐qPCR analysis for MGMT mRNA with actin as a control (mean ± SD, **P < 0.01, *n* = 3). E) Overexpression of MEN1 didn't influence the β‐Catenin mRNA level. MEN1‐ or negative control‐ overexpressing stable QGP‐1, Bon‐1 and HEK293 cells were subjected to RT‐qPCR analysis for β‐Catenin mRNA with actin as a control (mean ± SD, **P < 0.01, *n* = 3). F) Knockout of MEN1 didn't affect the MGMT mRNA level in PanNET cells. MEN1‐ or negative control‐ knocking out stable QGP‐1 and Bon‐1 cells were subjected to RT‐qPCR analysis for β‐Catenin mRNA with actin as a control (mean ± SD, **P < 0.01, *n* = 3). G) Overexpression of MEN1 reduced the MGMT promoter activity in PanNET cells. MEN1‐ or negative control‐ overexpressing stable QGP‐1, Bon‐1, and HEK293 cells were transfected with the reporter MGMT‐luc construct, and subjected to the determination of luciferase activity. TK‐Renilla luciferase plasmid was included in each transfection to normalize transfection efficiency (mean ± SD, **P < 0.01, *n* = 3). H) Knockout of MEN1 increased the MGMT promoter activity in PanNET cells. MEN1‐ or negative control‐ knocking out stable QGP‐1 and Bon‐1 cells were transfected with the reporter MGMT‐luc construct, and subjected to the determination of luciferase activity. TK‐Renilla luciferase plasmid was included in each transfection to normalize transfection efficiency (mean ± SD, **P < 0.01, *n* = 3). I) Immunohistochemistry (IHC) staining of mice islet cells using MEN1 and MGMT antibodies (*n* = 5). Scale bar for ×5 images: 5mm; Scale bar for ×100 images: 200 µm. J) Statistical analysis of MEN1 and MGMT expression in mice islet cells from Men1WT and Men1KO mice (**P < 0.01, *n* = 5). K) Immunohistochemistry (IHC) staining of human pancreatic neuroendocrine tumor tissues using MEN1, β‐Catenin and MGMT antibodies (*n* = 121). Scale bar for ×200 images: 100 µm. L) Statistical analysis showed a significant negative correlation between MEN1 and MGMT in PanNETs (*n* = 121, *P* < 0.001 was obtained using a Pearson χ2 test). M) Statistical analysis showed a significant negative correlation between MEN1 and β‐Catenin in PanNETs (*n* = 121, *P* < 0.001 was obtained using a Pearson χ2 test). These data were representative of three independent experiments. Data represented means, and error bars were standard deviations. Two‐sided t‐test.

To elucidate the relationship between MGMT and MEN1, we constructed stable MEN1‐overexpressing and MEN1‐knockout cells and examined the MGMT expression levels. As shown in **Figure** **4**A, compared with the vector negative control, MEN1 overexpression reduced the protein levels of MGMT and β‐Catenin. RT‒qPCR analysis also showed that overexpression of MEN1 decreased the mRNA level of MGMT (Figure 4B). Additionally, knockout of MEN1 induced the upregulation of MGMT expression according to WB and RT‒qPCR analysis (Figure 4C,D). Specifically, no significant changes in the mRNA levels of β‐Catenin after MEN1 overexpression or silencing were observed in the two PanNET cell lines (Figure 4E,F), indicating that MEN1 could regulate MGMT and β‐Catenin in different manners. Similarly, the MGMT promoter (−1.5 kb upstream)‐linked luciferase reporter assay showed that the transcription of MGMT could be inhibited by MEN1 overexpression and induced by MEN1 knockout (Figure 4G,H). In addition, we constructed pancreas‐specific MEN1‐KO mice (Pdx1‐Cre; MEN1^flox/flox^) to evaluate the relationship between MGMT and MEN1. Islet cell hyperplastic lesions were observed in MEN1‐KO mice (Pdx1‐Cre^ki/+^; MEN1^flox/flox^) compared with MEN1‐WT (wildtype) control mice (Pdx1‐Cre^+/+^; MEN1^flox/flox^) (Figure 4I). Importantly, according to IHC staining analysis, the levels of MGMT were significantly greater in hyperplastic islet cells from MEN1‐KO mice than in those from MEN1‐WT mice (Figure 4I,J). The negative correlation between MEN1 and MGMT was further confirmed by IHC staining of TMAs from 121 patients with PanNETs (Figure 4K,L). A negative correlation between MEN1 and β‐Catenin was also confirmed in patients with PanNETs (Figure 4K,M). Therefore, the data indicated that MEN1 controlled MGMT expression at the transcriptional level.

### 2.5 MEN1 Controlled the Transcriptional Activity of β‐Catenin and its Binding to the MGMT Promoter

**Figure 5:**
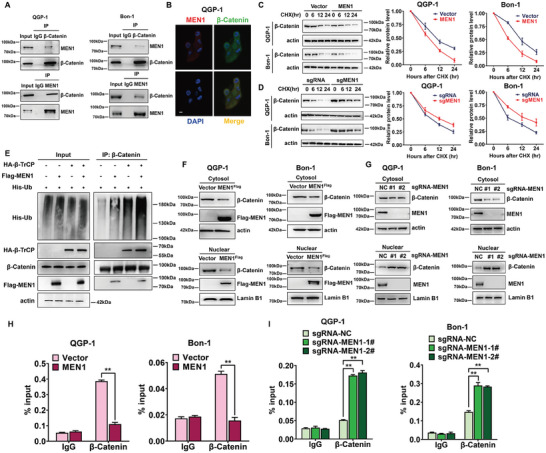
MEN1 controlled the transcriptional activity of β‐Catenin and its binding to the MGMT promoter. A) MEN1 interacted with β‐Catenin each other. Two PanNET cells were lysed and immunoprecipitated with anti‐β‐Catenin Ab or MEN1 Ab, and then subjected to immunoblotting using antibodies against MEN1 or β‐Catenin with IgG as a negative control. B) MEN1 colocalized with β‐Catenin in QGP‐1 cells. Localization of MEN1 (red) and β‐Catenin (green) in QGP‐1 cells was detected by double immunofluorescence labeling and confocal microscopy. The merged image with the orange signal represented their colocalization. Scale bar, 10 µm. C) Overexpression of MEN1 shortened the half‐life of β‐Catenin in PanNET cells. MEN1‐overexpressing stable Bon‐1 and QGP‐1 cells were pre‐treated with MG132 for 12 h. After that, cells were then treated by 50 µg mL^−1^ CHX for indicated time points, and subjected to immunoblotting using antibody against β‐Catenin with actin as a loading control. The protein level was quantified by densitometry. Two‐sided t‐test. D) Knock out of MEN1 extended the half‐life of β‐Catenin in PanNET cells. MEN1‐knocking out stable Bon‐1 and QGP‐1 cells were pre‐treated with MG132 for 12 h. After that, cells were then treated by 50 µg mL^−1^ CHX for indicated time points, and subjected to immunoblotting using antibody against β‐Catenin with actin as a loading control. The protein level was quantified by densitometry. Two‐sided t test. E) Overexpression of MEN1 increased the ubiquitination level of β‐Catenin. QGP‐1 cells were co‐transfected with Flag‐tagged MEN1 and other plasmids as indicated for 24 h, and then treated by 5 µm MG‐132 for 12 h, and subjected to immunoprecipitation using anti‐β‐Catenin antibody and immunoblotting using anti‐his antibody with actin as an input control. F) Overexpression of MEN1 inhibited the nuclear accumulation of β‐Catenin. MEN1‐overexpressing stable Bon‐1 and QGP‐1 cells were subjected to cytoplasmic and nuclear fractionation according to the manufacturer's protocol (P0027, Beyotime), and then subjected to immunoblotting using antibodies against β‐Catenin and Flag with β‐actin and Lamin B as cytoplasmic and nuclear fraction loading controls, respectively. G) Knockout of MEN1 promoted the nuclear accumulation of β‐Catenin. MEN1‐knocking out stable Bon‐1 and QGP‐1 cells were subjected to cytoplasmic and nuclear fractionation, and then subjected to immunoblotting using antibodies against β‐Catenin and Flag with β‐actin and Lamin B as cytoplasmic and nuclear fraction loading controls, respectively. H) Overexpression of MEN1 inhibited the binding of β‐Catenin to the MGMT promoter. ChIP assays were performed using anti‐IgG or anti‐β‐Catenin antibody in MEN1‐overexpressing stable Bon‐1 and QGP‐1 cells. The eluted DNA was subjected to RT‐qPCR with the specific primer of the MGMT promoter regions (**P < 0.01, *n* = 3). I) Knockout of MEN1 increased the binding of β‐Catenin to the MGMT promoter. ChIP assays were performed using anti‐IgG or anti‐β‐Catenin antibody in MEN1‐knocking out stable Bon‐1 and QGP‐1 cells. The eluted DNA was subjected to RT‐qPCR with the specific primer of the MGMT promoter regions (**P < 0.01, *n* = 3). These data were representative of three independent experiments. Data represented means, and error bars were standard deviations. Two‐sided t‐test.

Next, the mechanism underlying the transcriptional suppression of MGMT by MEN1 was explored. Previous studies reported that β‐Catenin is a transcription factor that plays an important role in promoting MGMT transcription,^[^ ^31^ ^]^ which is consistent with the findings of our study. In addition, MEN1 was reported to physically interact with β‐Catenin and inhibit its transcriptional activity. However, whether MEN1 controls MGMT transcription mainly through the regulation of β‐Catenin is still unclear. We first used an in vitro coimmunoprecipitation (CoIP) assay to validate the results of the current IP‐MS analysis in PanNET cells. As shown in **Figure** **5**A, endogenous MEN1 and β‐Catenin were able to bind to each other in the two PanNET cell lines, thereby confirming their interaction as identified via IP‐MS. In addition, the colocalization of MEN1 and β‐Catenin was also observed via cell immunofluorescence analysis (Figure 5B). Importantly, overexpression of MEN1 reduced the half‐life of β‐Catenin compared with that of the negative control after treatment with cycloheximide (CHX) (Figure 5C), while knockout of MEN1 significantly prolonged the half‐life of β‐Catenin compared with that of the control group after treatment with CHX (Figure 5D); these results indicated that MEN1 might regulate β‐Catenin degradation at the posttranslational level.

MEN1 has no ubiquitin E3 ligase activity, so how MEN1 controls the stability of β‐Catenin is unclear. β‐TrCP has been reported to be a typical E3 ubiquitin ligase that targets its substrate β‐Catenin for ubiquitination and degradation.^[^ ^34^ ^]^ We next examined whether MEN1 altered β‐Catenin ubiquitination by β‐TrCP. As shown in Figure 5E, overexpression of MEN1 notably increased the ubiquitination of β‐Catenin after β‐TrCP overexpression. The interaction between β‐TrCP and β‐Catenin was also enhanced by MEN1 overexpression, indicating their synergistic effects on the regulation of β‐Catenin degradation. Cao et al. reported that MEN1 could regulate the shuttling of β‐Catenin between the nucleus and the cytoplasm.^[^ ^8^ ^]^ Consistently, we found that the overexpression of MEN1 promoted the export of β‐Catenin from the nucleus and its subsequent degradation (Figure 5F). In contrast, MEN1 KO led to the nuclear accumulation of β‐Catenin (Figure 5G). A Chromatin immunoprecipitation (ChIP) assay showed that in the nucleus, β‐Catenin bound to the MGMT promoter and performed its transcriptional function. Similarly, we used a ChIP assay to show increased binding of β‐Catenin to the MGMT promoter in MEN1‐KO PanNET cells and decreased binding of β‐Catenin to the MGMT promoter in MEN1‐overexpressing cells (Figure 5H,I); these results suggest that MEN1 deficiency induced the binding of β‐Catenin to the MGMT promoter, while MEN1 overexpression inhibited the recruitment of β‐Catenin to the MGMT promoter, which resulted in suppressed MGMT transcription in PanNET cells.

### 2.6 MEN1 Controlled MGMT Transcription via the Regulation of β‐Catenin Activation in PanNETs

**Figure 6:**
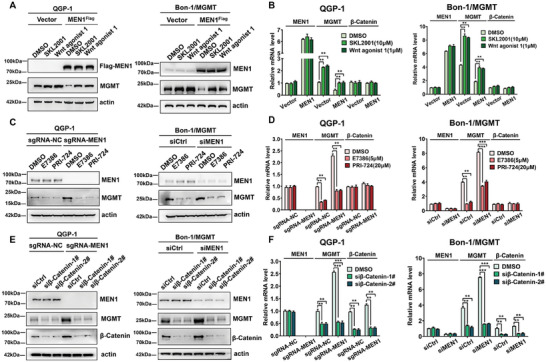
MEN1 controlled MGMT transcription via the regulation of β‐Catenin activation in PanNETs. A) The agonists of β‐Catenin rescued the reduction of the MGMT protein level by overexpression of MEN1. The expression of MGMT was examined by immunoblotting in MEN1‐overexpressing stable QGP‐1 cells, MEN1&MGMT‐coexpressing stable Bon‐1 cells and control stable cells treated with two agonists of β‐Catenin for 48 h. Actin was used as a loading control. B) The agonists of β‐Catenin rescued the reduction of the MGMT mRNA level by overexpression of MEN1. The levels of MGMT, MEN1, and β‐Catenin mRNA were examined by RT‐qPCR in MEN1‐overexpressing stable QGP‐1 cells, MEN1&MGMT‐coexpressing stable Bon‐1 cells and control stable cells treated with two agonists of β‐Catenin for 48 h. Actin was used as a loading control(**P < 0.01, *n* = 3). C) The antagonists of β‐Catenin counteracted the increase of the MGMT protein level by silencing of MEN1. MEN1‐knocking out stable QGP‐1 cells and control cells were treated with two antagonists of β‐Catenin for 48 h and subjected to immunoblotting. MGMT‐expressing stable Bon‐1 cells were transfected individually with siMEN1 or siControl for 24 h, followed by the treatment of two antagonists of β‐Catenin for 48 h, and then subjected to immunoblotting. Actin was used as a loading control. D) The antagonists of β‐Catenin counteracted the increase of the MGMT mRNA level by silencing of MEN1. MEN1‐knocking out stable QGP‐1 cells and control cells were treated with two antagonists of β‐Catenin for 48 h and subjected to RT‐qPCR for MGMT, MEN1, and β‐Catenin mRNA analysis. MGMT‐expressing stable Bon‐1 cells were transfected individually with siMEN1 or siControl for 24 h, followed by the treatment of two antagonists of β‐Catenin for 48 h, and then subjected to RT‐qPCR for MGMT, MEN1, and β‐Catenin mRNA. Actin was used as a loading control (***P < 0.001, **P < 0.01, *n* = 3). E) Knockdown of β‐Catenin inhibited the increase of the MGMT protein level by silencing of MEN1. MEN1‐knocking out stable QGP‐1 cells and control cells were transfected with siβ‐Catenin or siControl for 72 h and subjected to immunoblotting. MGMT‐expressing stable Bon‐1 cells were cotransfected with siMEN1 and siβ‐Catenin for 72 h and then subjected to immunoblotting. Actin was used as a loading control. F) Knockdown of β‐Catenin inhibited the increase of the MGMT mRNA level by silencing of MEN1. MEN1‐knocking out stable QGP‐1 cells and control cells were transfected with siβ‐Catenin or siControl for 72 h and subjected to RT‐qPCR for MGMT, MEN1 and β‐Catenin mRNA analysis. MGMT‐expressing stable Bon‐1 cells were cotransfected with siMEN1 and siβ‐Catenin for 72 h and then subjected to RT‐qPCR for MGMT, MEN1 and β‐Catenin mRNA analysis. Actin was used as a loading control (***P < 0.001, **P < 0.01, *n* = 3). These data were representative of three independent experiments. Data represented means, and error bars were standard deviations. Two‐sided t‐test.

To further evaluate the role of β‐Catenin in the transcriptional suppression of MGMT by MEN1, we examined whether β‐Catenin activation or inactivation could reverse the effects of MEN1 overexpression or knockdown on MGMT expression in PanNET cells. Two agonists of β‐Catenin, namely, SKL2001, and Wnt agonist 1 (also known as BML‐284 hydrochloride), which are coupled with the overexpression of MEN1, were administered to QGP‐1 cells and stable MGMT‐expressing Bon‐1 cells. As shown in **Figure** **6**A,B, compared with the negative control, the two β‐Catenin agonists reversed the effects of MEN1 overexpression on the mRNA and protein levels of MGMT. In contrast, two selective antagonists of β‐Catenin, namely, E7386, which was used in a phase I trial for patients with advanced solid tumors,^[^ ^35^ ^]^ and PRI‐724, which was used in a phase I / IIa study for patients with hepatitis C‐ or hepatitis B virus‐induced liver cirrhosis,^[^ ^36^ ^]^ were used. These agents decreased the mRNA and protein levels of MGMT in MEN1‐KO QGP‐1 cells and in MEN1‐knockdown Bon‐1 cells stably expressing MGMT (Figure 6C,D). Importantly, to further confirm this result, we knocked down β‐Catenin in these two cell lines and found that the mRNA and protein levels of MGMT were notably reduced compared with those in the negative control group (Figure 6E,F). Overall, our data suggested that β‐Catenin plays a key role in shaping the regulation of MGMT by MEN1, and the results further showed that either inhibition of β‐Catenin activity or the downregulation of β‐Catenin expression could reverse the transcriptional activation of MGMT caused by MEN1 deficiency. These results indicate that MEN1‐mediated regulation of MGMT transcription is dependent on the status of β‐Catenin in PanNETs.

### 2.7 Leucine 267 of MEN1 is Crucial for Regulation of the β‐Catenin‐MGMT Functional Axis and the Chemosensitivity of PanNET Cells to TMZ

**Figure 7:**
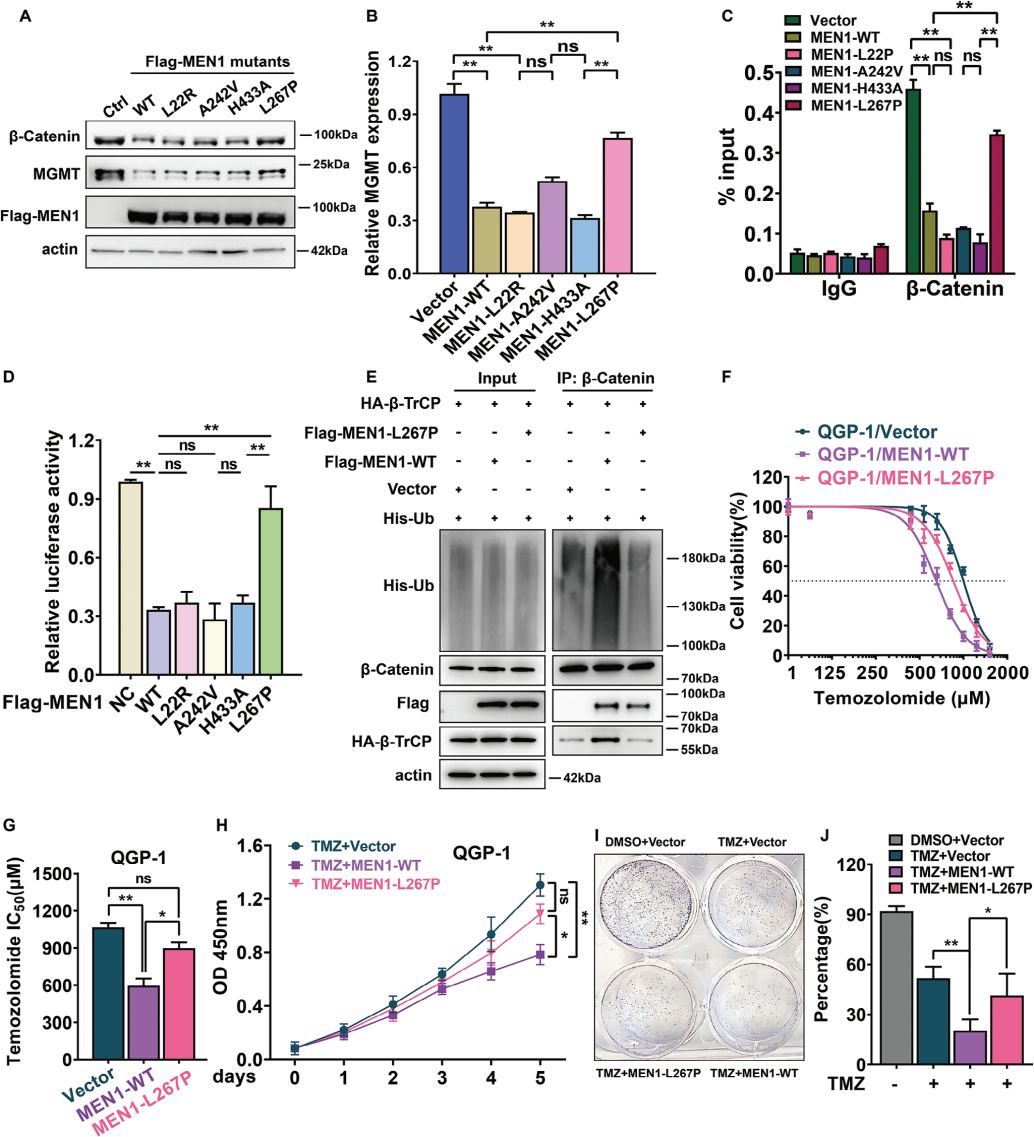
Leucine 267 of MEN1 is crucial for regulation of the β‐Catenin‐MGMT functional axis and the chemosensitivity of PanNET cells to TMZ. A) The L267P mutant of MEN1 failed to control the activation of the β‐Catenin‐MGMT axis. MEN1‐knocking out QGP‐1 cells stably re‐expressing different forms of MEN1 and negative control were subjected to immunoblotting using antibodies against β‐Catenin, MGMT and Flag with actin as a loading control. B) The Leu267 of MEN1 was required for regulation of MGMT mRNA level. MEN1‐knocking out QGP‐1 cells stably re‐expressing different forms of MEN1 and negative control were subjected to RT‐qPCR analysis for MGMT mRNA with actin as a loading control. C) The Leu267 of MEN1 was required for regulation of the binding of β‐Catenin to the MGMT promoter. ChIP assays were performed using anti‐IgG or anti‐β‐Catenin antibody in MEN1‐knocking out QGP‐1 cells stably re‐expressing different forms of MEN1 and negative control. The eluted DNA was subjected to RT‐qPCR with the specific primer of the MGMT promoter regions (**P < 0.01, *n* = 3). D) The Leu267 of MEN1 was required for regulation of the MGMT promoter activity. MEN1‐knocking out QGP‐1 cells stably re‐expressing different forms of MEN1 and negative control were transfected with the reporter MGMT‐luc construct, and subjected to the determination luciferase activity. TK‐Renilla luciferase plasmid was included in each transfection to normalize transfection efficiency (mean ± SD, **P < 0.01, *n* = 3). E) The Leu267 of MEN1 was required for regulation of the ubiquitination level of β‐Catenin. MEN1‐knocking out QGP‐1 cells stably re‐expressing indicated forms of MEN1 and negative control were cotransfected with HA‐β‐TrCP and His‐ubiquitin plasmids for 24 h, and then treated by 5 µm MG‐132 for 12 h, and subjected to immunoprecipitation using anti‐β‐Catenin antibody and immunoblotting using anti‐his antibody with actin as input control. F,G) The Leu267 of MEN1 was required for regulation of IC50 of TMZ. MEN1‐knocking out QGP‐1 cells stably re‐expressing indicated forms of MEN1 and negative control were treated with indicated dose of TMZ for 48 h, and then subjected to CCK8 assay (*P < 0.05, **P < 0.01, *n* = 3). H) The Leu267 of MEN1 was required for regulation of the tumor chemosensitivity to TMZ. MEN1‐knocking out QGP‐1 cells stably re‐expressing indicated forms of MEN1 and negative control were treated with 300 µm TMZ and then subjected to CCK8 assay for indicated time points (*P < 0.05, **P < 0.01, *n* = 3). I,J) The Leu267 of MEN1 was required for regulation of cancer cell proliferation in the presence of TMZ. 200 MEN1‐knocking out QGP‐1 cells stably re‐expressing indicated forms of MEN1 and negative control were treated individually with 300 µm TMZ or DMSO for 10 days and then subjected to colony formation assay (*P < 0.05, **P < 0.01, *n* = 3). These data were representative of three independent experiments. Data represented means, and error bars were standard deviations. Two‐sided t‐test.

MEN1 has the highest mutation frequency in PanNETs, and mutations in MEN1 result in partial or complete loss of physiological function. According to previous studies, we analyzed the levels of MGMT in MEN1‐KO QGP‐1 cells stably expressing wild‐type MEN1 or the well‐known MEN1 functional point mutants L22R, A242V, H433A and L267P. Among these variants, the L22R mutant has high histone methyltransferase‐reconstituting activity,^[^ ^37^ ^]^ the A242V mutant lacks this activity;^[^ ^38^, ^39^ ^]^ the H433A mutant cannot recognize H3K79me2^[^ ^40^ ^]^ and the L267P mutant lacks nuclear export activity.^[^ ^8^ ^]^ As shown in **Figure** **7**A, compared with the vector control, the L267P mutant, but not the other mutants, reversed the wild‐type MEN1 overexpression‐induced decrease in MGMT expression. Consistently, the L267P mutant also exhibited an increased mRNA level of MGMT compared with that of other forms of MEN1 (Figure 7B). In addition, ChIP analysis revealed that the binding of β‐Catenin to the MGMT promoter was increased with the L267P mutant but not with other forms of MEN1 (Figure 7C). We also observed that the L267P mutation restored the luciferase activity of the MGMT promoter (Figure 7D). These data indicated that MEN1 regulated the β‐Catenin‐MGMT signaling cascade in a manner that depended on the leucine 267 residue rather than its histone methyltransferase and “reading” H3K79me2 activities. Next, to explore the molecular mechanism by which the L267P mutant restored MGMT transcriptional recovery, we examined the level of phosphor‐β‐Catenin in MEN1‐ WT or ‐L267P mutant‐expressing cells. Consistent with the findings of previous studies.^[^ ^8^, ^41^ ^]^ WT‐MEN1 overexpression significantly increased the levels of phosphor‐β‐Catenin at Ser33, Ser47, Thr41, and Ser45 while L267P‐MEN1 overexpression failed to modulate the level of phosphorylated β‐Catenin (Figure S7, Supporting Information). Next, we further evaluated the ubiquitination level of β‐Catenin and its relationship with β‐TrCP in MEN1 wild‐type or mutant‐expressing cells. As shown in Figure 7E, the L267P mutant notably decreased β‐Catenin ubiquitination and attenuated the interaction between β‐Catenin and β‐TrCP compared with the wild‐type form, although there was no significant difference between the L267P mutant and the wild‐type form in terms of half‐life (Figure S8, Supporting Information) or interactions with β‐Catenin in QGP‐1 cells (Figure S9, Supporting Information). Importantly, we then examined the half‐maximal inhibitory concentration (IC_50_) of TMZ in QGP‐1 cells overexpressing the L267P mutant and the wild‐type form of MEN1 (Figure 7F). We found that PanNET cells overexpressing the L267P mutant had a higher inhibitory concentration (IC_50_) than those overexpressing the wild‐type protein (Figure 7G). Furthermore, the L267P mutant only slightly attenuated QGP‐1 cell growth in the presence of TMZ, and the suppressive effect was much weaker than that of wild‐type MEN1 upon treatment with TMZ (Figure 7H–J). Taken together, these data showed that the L267P mutant of MEN1 disrupted the transcriptional suppression of MGMT due to its inability to promote the nuclear export and degradation of β‐catenin; moreover, these data indicated that Leu 267 is a key residue in MEN1‐regulation of PanNET cell chemosensitivity to TMZ.

### 2.8 Alterations in the β‐Catenin‐MGMT Axis Re‐Sensitize PanNETs to TMZ

**Figure 8:**
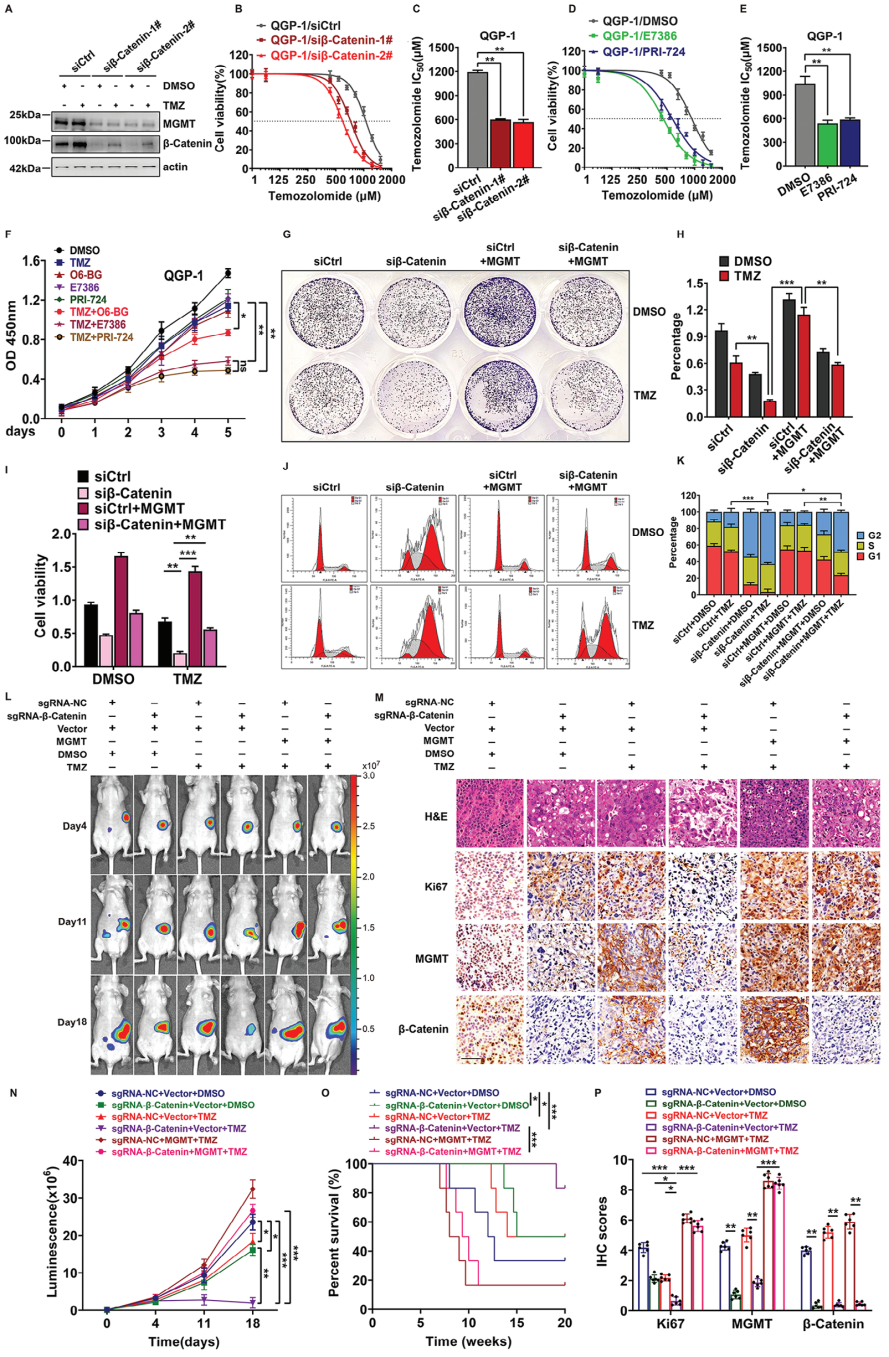
Alterations in the β‐Catenin‐MGMT axis re‐sensitize PanNETs to TMZ. A) Silencing of β‐Catenin disrupted the β‐Catenin‐MGMT signaling pathway upon the treatment of TMZ. QGP‐1 cells were transfected individually with siControl or siβ‐Catenin for 24 h, then treated with 300 µm TMZ or DMSO for 48 h, and subjected to immunoblotting using antibodies against β‐Catenin and MGMT with actin as a loading control. B,C) Knockdown of β‐Catenin significantly reduced IC50 of TMZ in QGP‐1 cells. QGP‐1 cells were transfected individually by siControl, siβ‐Catenin‐1# or siβ‐Catenin‐2# and treated with indicated dose of TMZ for 48 h, and then subjected to CCK8 assay (**P < 0.01, *n* = 3). D,E) The antagonists of β‐Catenin also reduced IC50 of TMZ in QGP‐1 cells. QGP‐1 cells were treated by two antagonists of β‐Catenin, combined with indicated dose of TMZ for 48 h, and then subjected to CCK8 assay (**P < 0.01, *n* = 3). F) The antagonists of β‐Catenin increased the tumor chemosensitivity to TMZ. QGP‐1 cells were treated by two antagonists of β‐Catenin, combined with 300 µm TMZ and then subjected to CCK8 assay for indicated time points (*P < 0.05, **P < 0.01, *n* = 3). G,H) Overexpression of MGMT rescued β‐Catenin depletion‐leading to inhibition of colony formation upon TMZ treatment in QGP‐1 cells. MGMT‐ or negative control‐ overexpressing stable QGP‐1 cells were transfected individually by siCtrl or siβ‐Catenin for 24 h, and treated with 300 µm TMZ for 10 days, and then subjected to colony formation assay (***P < 0.001, **P < 0.01, *n* = 3). I) Overexpression of MGMT rescued β‐Catenin depletion‐leading to inhibition of cell growth upon TMZ treatment in QGP‐1 cells. MGMT‐ or negative control‐ overexpressing stable QGP‐1 cells were transfected individually by siCtrl or siβ‐Catenin for 24 h, and treated with 300 µm TMZ for 48 h, and then subjected to CCK8 assay (***P < 0.001, **P < 0.01, *n* = 3). J,K) Overexpression of MGMT rescued β‐Catenin depletion‐leading to G2 phase arrest upon TMZ treatment in QGP‐1 cells. MGMT‐ or negative control‐ overexpressing stable QGP‐1 cells were transfected individually by siCtrl or siβ‐Catenin for 24 h, and treated with 300 µm TMZ for 48 h, and then subjected to PI staining and FACS analysis for cell‐cycle profile. L) Fluorescence of lesions generated in nude mice orthotopic pancreas individually injected with stably transfected QGP‐1 cells or control cells upon TMZ (40mg kg^−1^ day^−1^) intragastric administration for two weeks was examined in the indicated days, *n* = 6. M) Immunohistochemistry (IHC) staining of the lesions generated in mouse models using β‐Catenin, MGMT or Ki67 specific antibodies. (*n* = 6). Scale bar, 200 µm. N) Statistical analysis of luciferase intensities in mouse models at the indicated weeks. (mean ± SD, ***P < 0.001, **P < 0.01, *P < 0.05, *n* = 6). O) Survival of mice in the orthotopic pancreas‐seeded mouse models (mean ± SD, ***P < 0.001, **P < 0.01, *P < 0.05, *n* = 6, log‐rank test). P) Statistical analysis of IHC scores in mouse models (mean ± SD, ***P < 0.001, **P < 0.01, *P < 0.05, *n* = 6). These data were representative of three independent experiments. Data represented means, and error bars were standard deviations. Two‐sided t‐test.

Due to the much higher working concentration (IC_50_>10^3^ µm) and the potential cytotoxicity of TMZ when used to treat PanNETs, a new combination therapy with TMZ and other effective agents that target MGMT or the MGMT‐associated signaling pathway is of interest. However, multiple clinical trials using O6‐BG, which is a selective inhibitor of MGMT, to treat glioma revealed increased toxicity and more side effects. Most importantly, the outcome of patients with gliomas was not significantly improved after treatment with O6‐BG in those clinical trials,^[^ ^42^, ^43^, ^44^ ^]^ suggesting that the activation of the MGMT‐associated signaling pathway could compensate for the disruption of MGMT by O6‐BG. Thus, we explored whether targeting signaling pathway‐induced MGMT activation could re‐sensitize PanNET cells to TMZ. As shown in **Figure** **8**A, the knockdown of β‐Catenin via RNA interference downregulated the expression of proteins in the β‐Catenin‐MGMT functional axis of QGP‐1 cells upon TMZ treatment. We next determined the IC_50_ of TMZ and found that β‐Catenin knockdown notably decreased the IC_50_ of TMZ in QGP‐1 cells compared with that of the siRNA negative control (Figure 8B,C). Similarly, treatment with two inhibitors of β‐Catenin, namely, E7386, and PRI‐724, reduced the IC_50_ of TMZ (Figure 8D,E). Importantly, compared with O6‐BG and the control agent, a combination of E7386 and PRI‐724 with TMZ significantly suppressed QGP‐1 cell growth according to a CCK8 assay (Figure 8F).

Next, we examined whether the synergistic effect of TMZ and the suppression of β‐Catenin were dependent on the level of MGMT. As shown in Figure 8G,H, depletion of β‐Catenin coupled with TMZ inhibited QGP‐1 cell colony formation, but overexpression of MGMT reversed the suppression caused by β‐Catenin knockdown in the presence of TMZ. CCK8 analysis further showed that MGMT overexpression counteracted QGP‐1 sensitivity to TMZ by downregulating β‐Catenin expression (Figure 8I). Consistently, FACS analysis demonstrated that β‐Catenin knockdown in combination with TMZ treatment induced cell cycle arrest in the G2 phase, while simultaneous overexpression of MGMT antagonized this effect of the combination treatment on cell cycle arrest in the G2 phase in QGP‐1 cells (Figure 8J,K). Importantly, in the orthotopic pancreas‐seeded mouse models, the growth of tumors in the downregulated β‐Catenin expression group and the TMZ (40 mg kg^−1^ day^−1^) treatment group was inhibited compared with that in the control group (Figure 8L,N). Compared with the control group, the combination of β‐Catenin suppression and TMZ treatment led to significant synergistic inhibition of orthotopic pancreatic tumors and Ki67 expression in lesions, while the forced‐expression of MGMT strongly counteracted the orthotopic pancreatic tumor growth inhibition and the reduced expression of Ki67 induced by the synergistic effect of β‐Catenin depletion and TMZ treatment (Figure 8M,P). Similarly, the mice in the combination group had longer survivals that were markedly counteracted by the simultaneous overexpression of MGMT (Figure 8O). Taken together, these data indicate that the intervention of the β‐Catenin‐MGMT axis re‐sensitized PanNET cells to TMZ via the downregulation of MGMT expression.

## 3 Discussion

**Figure 9:**
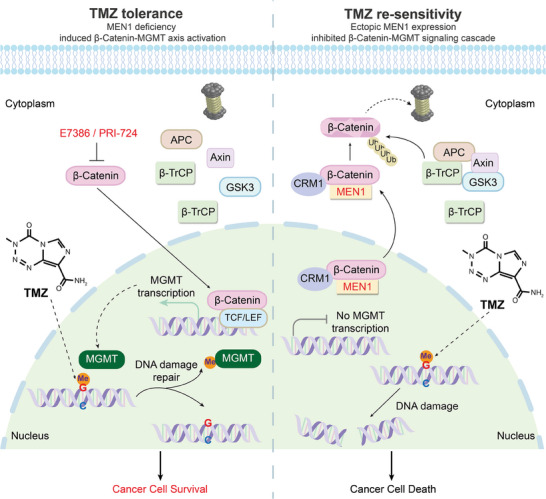
The proposed working model of this study. MEN1 deficiency induced the nuclear accumulation of β‐Catenin and the recruitment of β‐Catenin to the MGMT promoter, which promotes MGMT transcription and leads to PanNET cell growth and the chemosensitivity of tumor to TMZ. In turn, the restoration of MEN1 or the inhibition of β‐Catenin re‐sensitizes PanNETs to TMZ, thus providing a potential TMZ treatment‐based combination therapeutic strategy for treating patients with TMZ‐resistant PanNETs.

Despite the gradually increasing incidence of PanNETs, surgical resection is still the first choice for treating patients. However, due to the high heterogeneity and rarity of PanNETs, the molecular mechanisms underlying tumor heterogeneity and progression remain unclear. In addition, the drug resistance, recurrence and metastasis of PanNETs are still major clinical issues that clinicians face. A better understanding of the intrinsic mechanism and heterogeneity of PanNETs is needed to improve the outcomes of patients with PanNETs. TMZ, which is a first‐in‐class clinical chemotherapeutic drug, is widely used for the treatment of advanced PanNETs. However, due to the high level of MGMT, less than one‐third of patients benefit from this treatment. Moreover, the use of a much higher working dose of TMZ is associated with increased cytotoxicity. Based on these studies, we hope to elucidate the biological importance of MGMT and the mechanism that regulates its high expression; these studies will inform the development of possible effective intervention strategies for re‐sensitizing tumors to TMZ. In particular, in this study, we found that MEN1, which is the most important driver gene with PanNET characteristics, controlled MGMT transcription by regulating β‐Catenin activity. The Leu267 residue of MEN1 is crucial for controlling β‐Catenin‐MGMT axis activation. The biological importance of MGMT was also shown to play an oncogenic role in PanNETs. In addition, a negative correlation between MEN1 and MGMT was observed in pancreas‐specific MEN1‐KO mice and clinical specimens. The clinical implication was that inhibiting the β‐Catenin‐MGMT functional axis via β‐Catenin knockdown or the use of selective antagonists restored tumor chemosensitivity to TMZ. Overall, we showed for the first time that MEN1 deficiency induces the β‐Catenin‐MGMT signaling cascade and provided a new TMZ treatment‐based combination therapeutic strategy for disrupting this axis to treat patients with advanced PanNETs (**Figure** **9**, working model).

MEN1 is the most common gene that is mutated in PanNETs, and mutations in this gene lead to the loss of physiological function. Thus, MEN1 is thought to play a key role in tumorigenesis and cancer development.^[^ ^6^ ^]^ A transgenic mouse model showed that homozygous deletion of MEN1 results in death at E11.5‐13.5.^[^ ^45^ ^]^ Thus, conditional knockout mice, such as pancreas‐specific MEN1‐KO mice, are often used to assess the function of MEN1 after genetic alteration. For example, increased VEGF expression leads to abnormal vascular structures and vascularity with endocrine tumor occurrence in pancreas‐specific MEN1‐KO mice compared with the control group according to the Pdx1‐Cre‐Lox approach.^[^ ^46^ ^]^ Another study used the MPR (Men1^flox/flox^Pten^flox/flox^RIP‐Cre) and MPM (Men1^flox/flox^Pten^flox/flox^MIP‐Cre) systems to generate MEN1 and PTEN double KO mice and found that MEN1‐like tumors grew faster than the control group.^[^ ^47^ ^]^ Our previous study established pancreas‐specific MEN1‐KO mice and showed that compared with MEN1 wild‐type mice, MEN1‐KO exhibited mTOR signaling pathway activation and islet cell hyperplastic lesions.^[^ ^6^ ^]^ The data from these transgenic MEN1‐KO mice indicated that MEN1 performs multiple important biological functions in PanNETs, but the biological importance of MEN1 has not been fully elucidated. In addition, the function of MEN1 varies greatly among different types of tumors, microenvironments, cellular localizations and protein‒protein interactions (PPIs). For example, in contrast to that in solid tumors, MEN1 was shown to play a tumor suppressor role by assembling a histone methyltransferase complex with MLL1 that promotes the transcription of target genes (i.e., HOX) and cell proliferation in liquid tumors.^[^ ^48^ ^]^ Based on data presented in previous studies, our results suggested that MEN1 is shuttled between the cytoplasm and the nucleus in PanNETs. The translocation of MEN1 out of the nucleus further induces β‐Catenin degradation in the presence of β‐TrCP. L267P mutation of MEN1 largely reversed this effect of MEN1, which was consistent with previous findings.^[^ ^8^ ^]^ Importantly, MEN1 overexpression enhances the binding of β‐TrCP to β‐Catenin and increases the level of β‐Catenin ubiquitination, indicating that MEN1 facilitates either nuclear export or conformational changes of β‐Catenin and therefore exposes more binding surfaces of β‐Catenin to β‐TrCP.^[^ ^34^ ^]^ In contrast, the L267P mutant loses the two activities of MEN1 and therefore could be a promising target for the personalized treatment of patients with PanNETs, which prompted us to further screen and identify those with this point mutation via proteomics and other multiomics sequencing in many cohorts.

TMZ, which is an alkylating agent, produces methyl adducts in DNA bases (i.e., O^6^‐MG) to cause DNA mismatches and double‐stranded breaks, ultimately leading to cell cycle arrest and tumor cell death. TMZ is abundantly used in the standard treatment of various tumors,^[^ ^14^, ^15^ ^]^ including PanNETs and glioblastoma (GBM). However, TMZ resistance and toxicity are still the main clinical problems. A recent study reported that hypoxanthine phosphoribosyl transferase 1 (HPRT1) is identified to metabolize TMZ to activate AMPK (Adenosine 5‘‐monophosphate (AMP)‐activated protein kinase) and RRM1(the catalytic subunit of ribonucleotide reductase) and increase the production of dNTPs that supplement the damaged DNA induced by TMZ in glioblastoma. 6‐Mercaptopurine (6‐MP), which is an inhibitor of HPRT1, sensitizes brain tumor cells to TMZ treatment in mice, indicating that HPRT1 is a new target for overcoming TMZ resistance.^[^ ^49^ ^]^ Previous studies have shown that TMZ treatment can activate the mTOR pathway and the PI3K/Akt/GSK‐3/β‐Catenin signaling cascade, thus leading to the evasion of apoptosis and the glioma chemoresistance to TMZ.^[^ ^50^, ^51^ ^]^ Another mechanism that explains TMZ resistance is that an enhancer located between the promoters of the Ki67 and MGMT genes is activated in TMZ‐resistant patient‐derived xenograft (PDX) models, which promotes Ki67 and MGMT expression and TMZ resistance in glioblastoma.^[^ ^33^ ^]^ Similarly, we showed a novel unique cascade with PanNET characteristics that controls the transcription of MGMT and tumor chemosensitivity to TMZ. Therefore, in the presence of no MGMT mutations observed in PanNETs, we speculated that TMZ resistance was mainly attributed to two causes: one was the activation of MGMT, including a high level of MGMT expression (i.e., promoter methylation or transcription) and the activation of MGMT‐associated upstream signaling pathways (i.e., the Wnt/β‐Catenin or p53 pathway); the other was the effect induced by TMZ metabolites, such as the activation of DNA damage repair systems (i.e., mismatch repair or base excision repair).

The problem of TMZ‐resistance in PanNETs is multifaceted. Various key determinants, including tumor burden and growth kinetics, tumor heterogeneity, tumor microenvironment and undruggable cancer drivers, could lead to drug resistance through a variety of mechanisms. According to the time of tumor evolution, chemoresistance is divided into two main types: intrinsic‐ or acquired resistance. Intrinsic factors exist before the treatment and accompany the tumor at all times. For example, the constitutive activation of intrinsic signaling pathways like Wnt‐β‐Catenin, PI3K/Akt, and NF‐κB axis affected the chemo‐resistance of patients with Pancreatic Ductal Adenocarcinoma (PDAC) by reducing the distribution and the concentration of gemcitabine at the tumor site.^[^ ^52^, ^53^ ^]^ Acquired factors are generated after the therapy and induced by the drug. The reasons for this form of resistance are the changes in DNA repair, the alterations in the tumor microenvironment and the mutations of drug targets. It suggested that the underlying reason for TMZ resistance in tumor proliferation with high MGMT levels is attributed to a combinational effect from the inherent genetic mutations like MEN1, the activation of intrinsic pathways like Wnt‐β‐Catenin, the newly acquired DNA bases in cell cycle and even the changes in the tumor microenvironment. By contrast, despite patients with low levels of MGMT expression having a favorable outcome compared with those with high levels of MGMT in our study, TMZ resistance is still observed in glioblastoma (GBM) cells with MGMT negative.^[^ ^54^ ^]^ The possible mechanism for the chemoresistance of gliomas is that β‐catenin, the phosphor‐Akt and the phosphor‐PRAS40 are increased, thus inducing Epithelial‐to‐mesenchymal transition (EMT) in TMZ‐resistant GBM cells, indicating that mechanisms other than mediated by MGMT expression lead to drug resistance from the cell‐type specific context. Specifically, TMZ‐induced DNA fragments are more likely to lead to antigen neogenesis, which is associated with sensitivity to immune checkpoint inhibitor (ICI) treatment.^[^ ^55^ ^]^ Thus, it is not difficult to understand that O6‐BG, which is a specific inhibitor of MGMT, decreases MGMT expression and theoretically increases tumor sensitivity to TMZ via inhibition of MGMT, but actually, it does not exhibit a significant improvement in the outcome of patients with gliomas in clinical trials.^[^ ^42^, ^43^ ^]^ Therefore, our future studies will focus on the crosstalk between the MGMT‐associated signaling cascade and the DNA damage repair (DDR) signaling pathway induced by TMZ treatment, as well as the relationship between ICIs and TMZ chemotherapy.

In conclusion, we identified the oncogenic role of MGMT in PanNETs and elucidated the mechanism underlying the regulation of its high expression. MEN1 deficiency induced the nuclear accumulation of β‐Catenin and the recruitment of β‐Catenin to the MGMT promoter, which promotes MGMT transcription and leads to cancer cell growth and disruption of the TMZ response. The Leu267 residue of MEN1 is critical for regulating activation of the β‐Catenin‐MGMT axis. Interference with the β‐catenin‐MGMT signaling cascade re‐sensitized PanNET cells to TMZ treatment. Overall, our study revealed a new mechanism by which MGMT expression is regulated by MEN1 in PanNETs and provided a potential combinational therapeutic strategy to support decision‐making in the treatment of patients with TMZ‐resistant PanNETs.

## 4 Experimental Section

### Study Population

A total of 121 patients who were clinically diagnosed with PanNETs and receiving surgical treatment were enrolled and followed up at Fudan University Shanghai Cancer Center from June 2012 to January 2022; written informed consent forms were obtained from the Institutional Research Ethics Committee (No.2105235) and signed by the patients. The studies were conducted in accordance with the Declaration of Helsinki, and the studies were approved by the institutional review board. Seventy paired cancer tissues and adjacent tissues were used to construct tissue microarrays (TMAs). Progression‐free survival (PFS) was defined as the time from the date when a patient underwent surgery to the date of disease progression, according to the published methods.^[^ ^6^ ^]^ The clinicopathological characteristics of the patients are shown in Table S1 (Supporting Information).

### Cell Culture and Reagents

The human PanNET cell line Bon‐1 was donated by Professor Martyn Caplin (Professor of Gastroenterology & Gastrointestinal Neuroendocrinology Centre for Gastroenterology, Royal Free Hospital, London) and grown in DMEM/Nutrient Mixture F‐12 medium supplemented with 10% fetal bovine serum (Biochrom, Holliston, MA) and 1% penicillin–streptomycin solution in a humidified atmosphere of 5% CO_2_ and 95% air at 37 °C. The human PanNET cell line QGP‐1 was obtained from Shanghai Zhong Qiao Xin Zhou Biotechnology Co., Ltd.(Shanghai, China), authenticated by short tandem repeat analysis and grown in RPMI 1640 medium supplemented with 10% fetal bovine serum (Biochrom, Holliston, MA) and 1% penicillin–streptomycin solution in a humidified atmosphere of 5% CO_2_ and 95% air at 37 °C. The human PanNET cell line SPNE1 was generated from the operative specimen of a primary pancreatic tissue of a 44‐year‐old female patient with G3‐differentiated NET and grown in DMEM/F12 supplemented with GlutaMAX (both Thermo Fisher Scientific, Waltham, MA, USA) supplemented with 10% fetal bovine serum (Biochrom, Holliston, MA) and 1% penicillin–streptomycin solution in a humidified atmosphere of 5% CO_2_ and 95% air at 37 °C that referred to the previous study.^[^ ^56^ ^]^ All the other cell lines were obtained from the ATCC and grown under standard conditions. Temozolomide (TMZ; SJ‐MX0064) and O6‐Benzylguanine (O6‐BG; SJ‐MX3716) were purchased from Shandong Sparkjade Biotechnology Co., Ltd.(Shandong, China), SKL2001 (S8320), Wnt agonist 1(BML‐284 hydrochloride; S8178), E7386 (E1348), PRI‐724 (ICG‐001 analog; S8968), and cycloheximide (CHX; S7418) were purchased from selleck.cn and used for in vitro studies followed by the manufacturer's instruction.

### Western Blot and CHX‐Chase Analysis

Cell lysates were prepared for Western blot analysis using antibodies against MEN1 (D262984, BBI), MGMT (A11151, ABclonal), β‐Catenin (A19657, A20221, ABclonal), Phospho‐β‐Catenin‐S33/S37/T41(AP0524, ABclonal), Phospho‐β‐Catenin‐S45(AP0580, ABclonal), BAD(A19595, ABclonal), BAX(A0207, ABclonal), Wee1(#13 084, Cell signaling), p27(#3686, Cell signaling), p21(#2947, Cell signaling), Cleaved PARP (#94 885, Cell signaling), Flag(AE005, ABclonal), HA(AE008, ABclonal), His(AE003, ABclonal). Lamin B1(A16909, ABclonal), and β‐actin (AC038, ABclonal) were used as the loading control. To determine the half‐life of β‐Catenin, cells were treated with 50 mg mL^−1^ CHX (Selleck) for the indicated time followed by the previous study.^[^ ^6^ ^]^

### TMA and Immunohistochemical (IHC) Staining

The tumor tissues and paired adjacent normal tissues were collected and re‐embedded in TMA blocks, and then the samples were subjected to a series of routine processes, including deparaffinization, rehydration, antigen retrieval, and removal of endogenous peroxidase. The slides were further blocked using 5% normal goat serum and incubated with primary antibodies. Antibodies against MEN1 (D262984, BBI), β‐Catenin(ab32572, Abcam), and MGMT (ab39253, Abcam) were used at a concentration of 1:200. The DAB‐stained slides were observed under a microscope, and images were captured and further quantitatively classified according to staining intensity and area of positive staining. As previously described,^[^ ^6^ ^]^ the specimens were divided into five groups: the weakest group (±), weak group (+), medium group (+ +), strong group (+ + +), and strongest group (+ + + +). The ±, +, and + + groups were further subdivided into the low‐expression groups and the + + + and + + + + groups into the high‐expression groups.

### Transcriptome Sequencing

Total RNA was used as input material for the RNA sample preparations. Briefly, mRNA was purified from total RNA using poly‐T oligo‐attached magnetic beads. To preferentially select cDNA fragments 370–420 bp in length, the library fragments were purified with the AMPure XP system. The clustering of the index‐coded samples was performed on a cBot Cluster Generation System using TruSeq PE Cluster Kit v3‐cBot‐HS (Illumina) according to the manufacturer's instructions. After cluster generation, the library preparations were sequenced on an Illumina NovaSeq platform, and 150 bp paired‐end reads were generated. Clean data (clean reads) were obtained by removing reads containing adapters, reads containing poly‐N and low‐quality reads from the raw data. The index of the reference genome was built using HISAT2 v2.0.5, and paired‐end clean reads were aligned to the reference genome using HISAT2 v2.0.5. Differential expression analysis of two conditions/groups (two biological replicates per condition) was performed using the DESeq2 R package (1.20.0). Genes with an adjusted P value ≤0.05, as determined by DESeq2, were considered to be differentially expressed.

### Cell Proliferation and Clonogenic Survival Assays

For the cell proliferation assay, Bon‐1 or QGP‐1 cells were seeded in 96‐well plates at 2500 cells per well in triplicate, and then, the cells were cultured for the indicated durations. Cell viability was determined with the Cell Counting Kit‐8 kit (C0042, Beyotime), ATPlite Luminescent (40210ES10, Yeasen), and EdU with Alexa Fluor 488 Cell proliferation kit (C0071S, Beyotime) following the manufacturer's instructions. For the clonogenic assay, cells were seeded in 6‐well plates in triplicate and cultured for 10 days. Then, colonies were fixed with 4% paraformaldehyde solution, stained with crystal violet, and counted under a microscope. Representative results of three independent experiments with consistent trends were presented.

### Ubiquitination Assay

To analyze β‐Catenin ubiquitination, PanNET cells were cotransfected with plasmids expressing wild‐type or mutant MEN1, HA‐β‐TrCP, His‐Ub or the negative control. The cells were then harvested and lysed using NP‐40 lysis buffer (P0013F, Beyotime) on ice. Afterward, the cell lysates were incubated with 5 µL of anti‐β‐Catenin (ab32572, Abcam) Ab or rabbit IgG control Ab at 4 °C overnight. Twenty microliters of protein A+G agarose (Santa Cruz Biotechnology) were then added and incubated for 1 h at 4 °C. The beads were washed with NP‐40 lysis buffer several times and subjected to IB.

### Propidium Iodide Staining and FACS Analysis

The cell cycle distribution was determined by propidium iodide (PI) staining and FACS analysis according to the published methods.^[^ ^57^ ^]^ In brief, cells were harvested and fixed in 70% ethanol solution at 20 °C overnight, and then, the cells were stained with PI (36 mg mL^−1^; Sigma) supplemented with RNase (10 mg mL^−1^; Sigma) at 37 °C for 30 min. Finally, the cell cycle distribution was analyzed by CyAn ADP (Beckman Coulter). The data were analyzed using ModFit LT software (Verity Software House). Representative results of three independent experiments with consistent trends were presented.

### Chromatin Immunoprecipitation (ChIP)‐PCR

According to the manufacturer's instructions (#9003, Cell signaling), PanNET cells were fixed with 1% formaldehyde in PBS and incubated in swelling buffer to prepare to extract the nuclei. Chromatin was then sheared by sonication and incubated with 2 µg of anti‐β‐catenin antibody (ab32572, Abcam) and 25 µl ChIP‐Grade Protein G Magnetic Beads (#9006, Cell signaling) overnight at 4 °C. The beads were successively washed with 1 ml sonication buffer, 1 ml high salt buffer, and 1 ml LiCl wash buffer three times. Chromatin was eluted and purified using a PCR purification kit (28 104, Qiagen). Enriched chromatin regions were characterized by real‐time quantitative PCR (RT‐qPCR). The primers of MGMT promoter regions used for ChIP referred to a previous study.^[^ ^58^ ^]^

### Luciferase Reporter Assay

Plasmids encoding a firefly luciferase reporter gene under the control of ≈1.5 kb of the distant MGMT promoter were transfected into PanNET cells along with a pRL‐SV40 normalization reporter plasmid using Lipofectamine 2000 (Invitrogen). Afterward, the cells were harvested in passive lysis buffer (Promega), and 15 µl of cell lysates were transferred to 96‐well LumiNunc plates (Thermo Scientific). Firefly luciferase and Renilla luciferase intensities were measured using D‐luciferin buffer and coelenterazine buffer, respectively, in the CentroXS LB960 lumiometer (Berthold Technologies).

### Cell Fractionation

According to the manufacturer's instructions (#9038, Cell signaling), PanNET cells were resuspended by 500 ul CIB (Cytoplasm Isolation Buffer) and incubated on ice for 5 min. After centrifuge, the supernatant was collected for the cytoplasmic fraction and subjected to IB. The pellet was then resuspended in 250 µl of CyNIB (Cytoskeleton/Nucleus Isolation Buffer) and sonicated for several times. After centrifuge, the supernatant was collected for the nuclear fraction and subjected to IB. Lamin B1(A16909, ABclonal) and β‐actin (AC038, ABclonal) were used as the loading control in the cytoplasmic fraction and the nuclear fraction, respectively.

### RNA Interference

Bon‐1 or QGP‐1 cells were transfected with siRNA oligonucleotides using Lipofectamine 3000 reagent (Life Technologies, Invitrogen) according to the manufacturer's instructions. The sequences of siRNA were as follows: For MEN1: si MEN1‐1#: 5′‐ GGGAAGACGAGGAGAUCUACA‐3′; siMEN1‐2#: 5′‐ GAAGGUCUCCGAUGUCAUAUG‐3′; For β‐Catenin: siβ‐Catenin‐1#: 5′‐ AAUCACAAACCUUGAGUAGCC −3′; siβ‐Catenin‐2#, 5′‐ UCACCUCGUGGUACCUGAAUU −3′; For SP‐1: siSP‐1: 5′‐GCAACAUCAUUGCUGCUAU −3′; For AP‐1: siAP‐1: 5′‐ AGCGGAGACAGACCAACUAGA −3′; For AP‐2: siAP‐2: 5′‐ GCUGGGCACUGUAGGUCAAUC −3′; For p65: sip65: 5′‐ CUUCCAAGUUCCUAUAGAA −3′; For HIF1α: siHIF1α: 5′‐ AAAAACUUCAGACUCUUUGCU −3′; For p300: sip300: 5′‐ CUGUCAGAAUUGCUGCGAUC −3′; For C‐JUN: siC‐JUN: 5′‐ GCAUGGACCUAACAUUCGA −3′; For control scrambled siRNA, siControl: 5′‐UUCUCCGAACGUGUCACGUTT‐3′. All the above siRNAs were ordered from GenePharma (Shanghai, China).

### LC/MS‐MS Analysis

For coimmunoprecipitation (Co‐IP)/MS, Flag‐β‐Catenin‐expressing stable QGP‐1 cells were lysed in pre‐chilled NP‐40 lysis buffer (Beyotime) on ice. The whole cell extracts were incubated with 5 µL anti‐Flag(ABclonal) Ab or rabbit IgG control Ab for 2 h followed by ultracentrifugation and 1 h incubation with 10 µL Protein A+G agaroses (Santa Cruz Biotechnology) in 4 °C. Beads were washed 3 times with NP‐40 lysis buffer and subjected to SDS‐PAGE gel separation. Gels stained with silver staining kit (Beyotime) were cut off and then destained using destain buffer (Beyotime). Immunocomplexes were digested with trypsin and identified on a Fusion Lumos Mass Spectrometer (Thermo Fisher Scientific). Raw data were searched against the human protein RefSeq database in Firmiana.

### Mice

TO generate Men1‐knockout (KO), mice in which the Men1 alleles were flanked by loxP sites (Men1^flox/flox^) were crossed to Pdx1‐Cre transgenic mice (strain NO. T004860, GemPharmatech, Nanjing, China) to yield Pdx1‐Cre^ki/+^; Men1^flox/+^ heterozygous mice. Pdx1‐Cre^ki/+^; Men1^flox/+^ heterozygous mice were identified and further bred to generate the desired Men1 KO genotypes, namely, the Pdx1‐Cre^ki/ki^; Men1^flox/flox^ genotype, the Pdx1‐Cre^ki/+^; Men1^flox/flox^ genotype, and the Pdx1‐Cre^+/+^; Men1^flox/flox^ control genotype. All the mice were genotyped by PCR using DNA that was extracted from the tail as previously described.^[^ ^6^ ^]^ Primers were used for Pdx1‐Cre (Forward, 5′‐GGGCAGTCTGGTACTTCCAAGCT‐3′; Reverse, 5′‐TTCTGGGCTATACAAGCATCTGC‐3′) and Men1 floxed alleles (Forward, 5′‐TAAACCTTGTGTGGTGGGGCAG‐3′; Reverse, 5′‐ CTTTGTCCTTAGTCAAGCCCGTG −3′), respectively.

To establish orthotopic pancreatic neuroendocrine tumor models, 5‐week‐old female athymic BALB/c nude mice were ordered from the Shanghai Experimental Animal Center (Shanghai, China). The prior application was approved by an internal animal protocol review committee. Briefly, subconfluent cultures of luciferase‐expressing Bon‐1 and QGP‐1 cells were collected and examined for viability. The cells were then resuspended in ice‐cold PBS, respectively and were mixed gently to produce a homogeneous suspension. BALB/c nude mice were anesthetized by intraperitoneal injection of 1% pentobarbital (50 mg kg^−1^). After local disinfection, the abdominal cavity was opened by a 1.0 cm longitudinal incision in the left upper quadrant. The spleen was lifted to identify the tail of the pancreas. Next, fifty microliters of ice‐cold PBS containing 1 × 10^6^ cells were then slowly injected into the pancreatic parenchyma by a prechilled insulin syringe with an ice‐cold 27‐gauge needle. The needle was kept on the injection site for 15 s to prevent the leakage of cells. The pancreas and spleen were slowly put back into the abdominal cavity, and the cavity was sutured using a two‐layer running silk suture. In the orthotopic pancreas injection, the firefly luciferase intensities of implantation lesions were measured every week by staining with D‐luciferin (150 mg kg^−1^, Xenogen, Alameda, CA) in an in vivo imaging system (Xenogen). The mice were sacrificed in a carbon dioxide chamber at the end of the study and processed for routine histological examination. Antibodies against Ki67(ab15580, Abcam), MEN1 (ab92443, Abcam), β‐Catenin (ab32572, Abcam), and MGMT (MA3‐16537, Thermo Fisher Scientific) were used for mouse immunohistochemical (IHC) staining. All the procedures were performed in accordance with the NIH Guide for the Care and Use of Laboratory Animals, and the Institutional Animal Care and Use Committee (IACUC) approved these studies (No. FUSCC‐IACUC‐S2022‐0392).

### Statistical Analysis

All data were presented as the mean ± SD. Two‐tailed Student's t‐test was used to compare parameters between two groups, and the χ^2^ test was used to assess the correlation between two categorical variables. Kaplan–Meier survival function and log‐rank test were used to evaluate the PFS of patients. The statistical significance of differences between groups was analyzed by GraphPad Prism 9 software. Three levels of significance (*, *P* < 0.05; **, *P* < 0.01; ***, *P* < 0.001) were applied, and a P value < 0.05 was considered to indicate statistically significant differences.

### Ethics Approval and Consent to Participate

The informed consent of patients and approval was gained from the Clinical Research Ethics Committee of Fudan University Shanghai Cancer Center. All animal experimental procedures were performed in accordance with the protocols approved by the Institutional Animal Care and Research Advisory Committee of Fudan University Shanghai Cancer Center.

## Conflict of Interest

The authors declare no conflict of interest.

## Author Contributions

J.X., X.L., F.W., W.Z., and X.X. contributed equally to this work. J.X. performed Conceptualization, data curation, software, funding acquisition, validation, investigation, methodology, wrote the original draft, project administration, and provided resources. X.L. and W.Z. performed methodology and project administration. F.W. and Y.W. performed project administration. X.X.,Y.Q., and X.Y. performed supervision and funding acquisition. Z.Y., Y.Z., and C.Z. provided resources. Q.Z. provided resources and performed project administration. D.J. and G.F. performed methodology. X.C. performed funding acquisition and provided resources. J.C. performed Funding acquisition. S.J. performed conceptualization, supervision, and funding acquisition.

## Supporting information

Supporting Information

## Data Availability

The data that support the findings of this study are available from the corresponding author upon reasonable request.
