# Supplementary material for: MEN1 Deficiency‐Driven Activation of the β‐Catenin‐MGMT Axis Promotes Pancreatic Neuroendocrine Tumor Growth and Confers Temozolomide Resistance
Source: Adv Sci (Weinh). 2024 Jul 23;11(35):2308417. doi: 10.1002/advs.202308417 (PMC11425246; doi:10.1002/advs.202308417)
Supplement: Supplementary file 1 — Supporting Information [file ADVS-11-2308417-s001.docx]

Supporting information

**B**

**A**


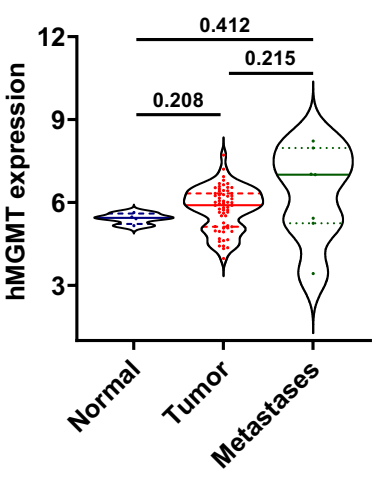

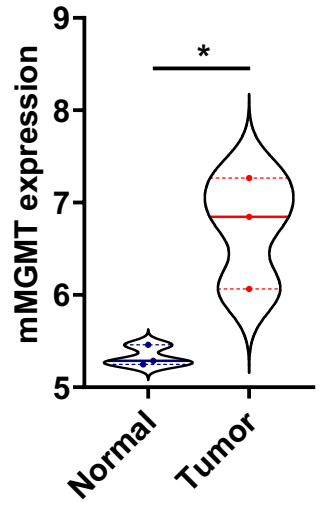


**Figure S1.** GEO RNA-Seq database demonstrated MGMT expression levels in mouse pancreatic neuroendocrine tumor (A, left, GSE248606) and human pancreatic neuroendocrine tumor (B, right, GSE73338). Gene expression levels of MGMT were higher in pancreatic cancer tissues (n = 3) than adjacent normal tissues (n = 3), *p < 0.05.


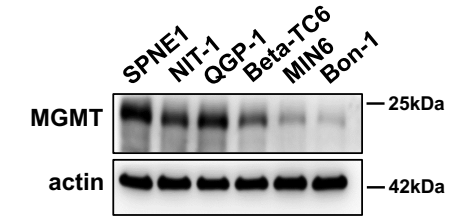


**Figure S2.** Immunoblot analysis of MGMT expression in the indicated pancreatic neuroendocrine tumor cell lines. NIT-1, Beta-TC6 and MIN6 were mice cell lines and SPNE1, QGP-1 and Bon-1 were human cell lines. Actin was used as a loading control.

**A**


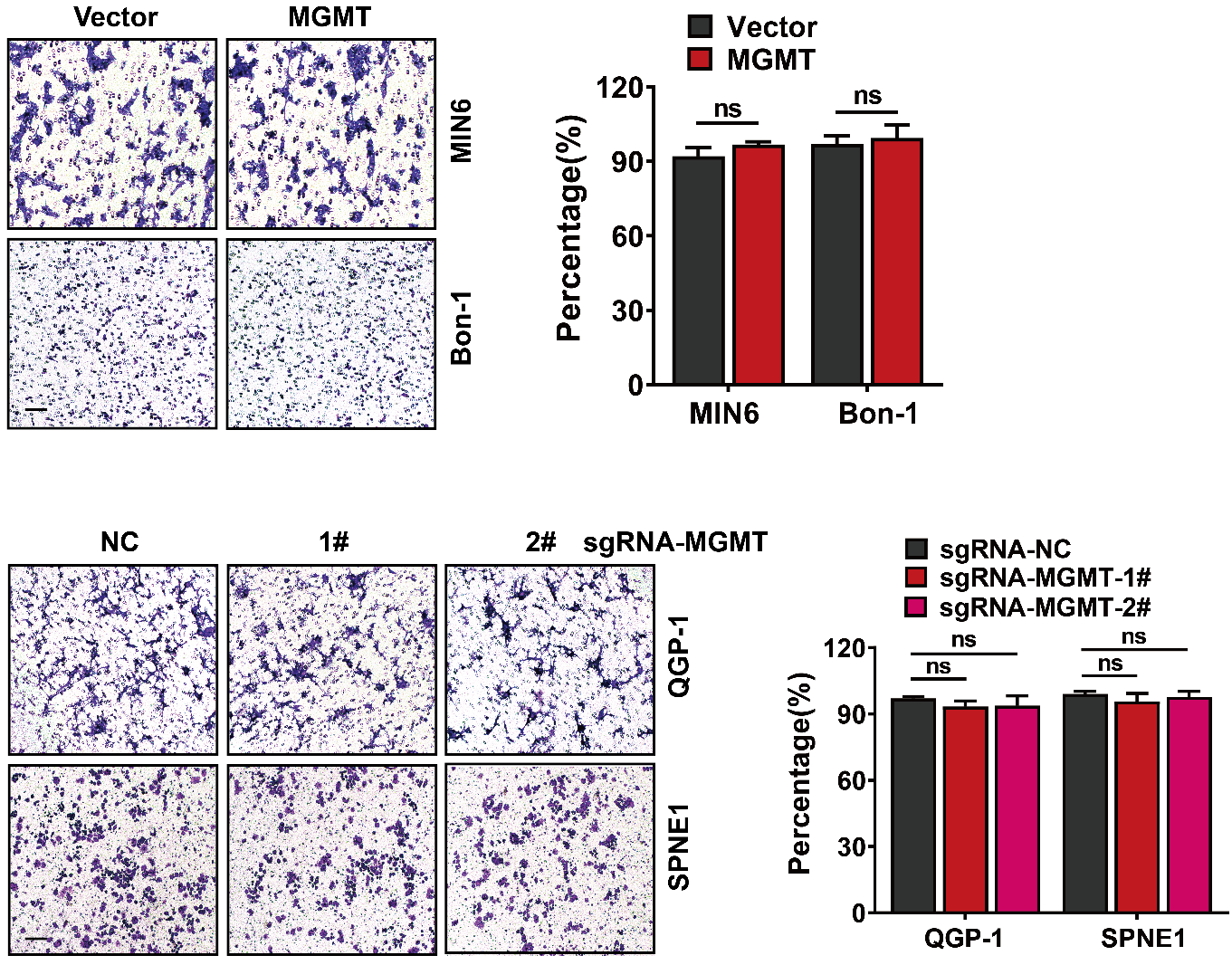


**B**

**Figure S3. MGMT up-/down- regulation did not affect PanNET cell migration.** MGMT overexpressing stable highly-invasive MIN6 or Bon-1 cells, and MGMT knockout stable highly-invasive QGP-1 and SPNE1 cells were suspended in serum-free medium and added into transwell chamber for 36 hours, and cells passed through the polycarbonate membrane (12-mm pore size; Millipore)were counted under a Leica microscope (n=3). Scale bar, 100μm.


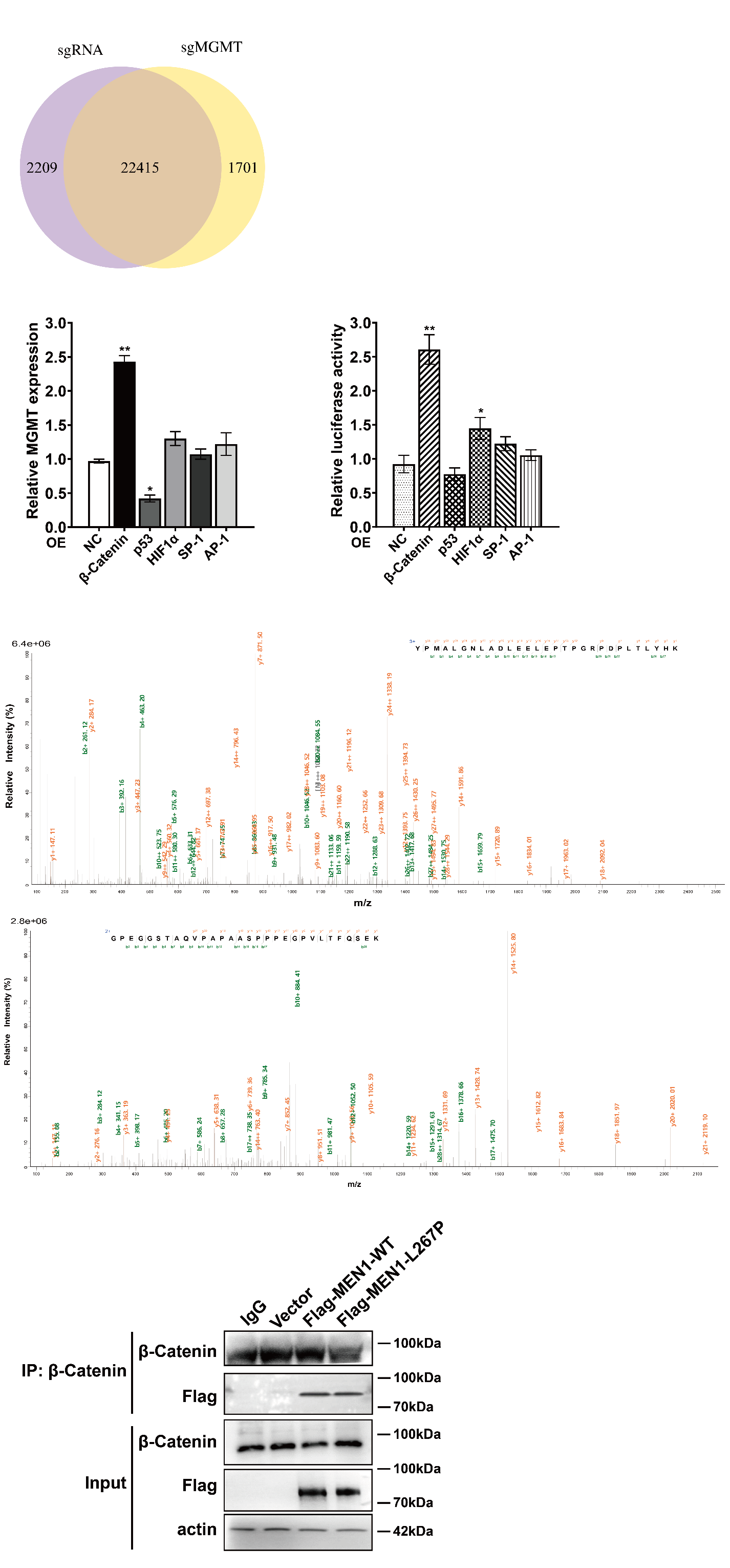


**Figure S4.** Venn diagram showed that a total of 1701 genes were upregulated and 2209 genes were downregulated in stable sgRNA-MGMT vs. sgRNA-negative control (NC) cells.


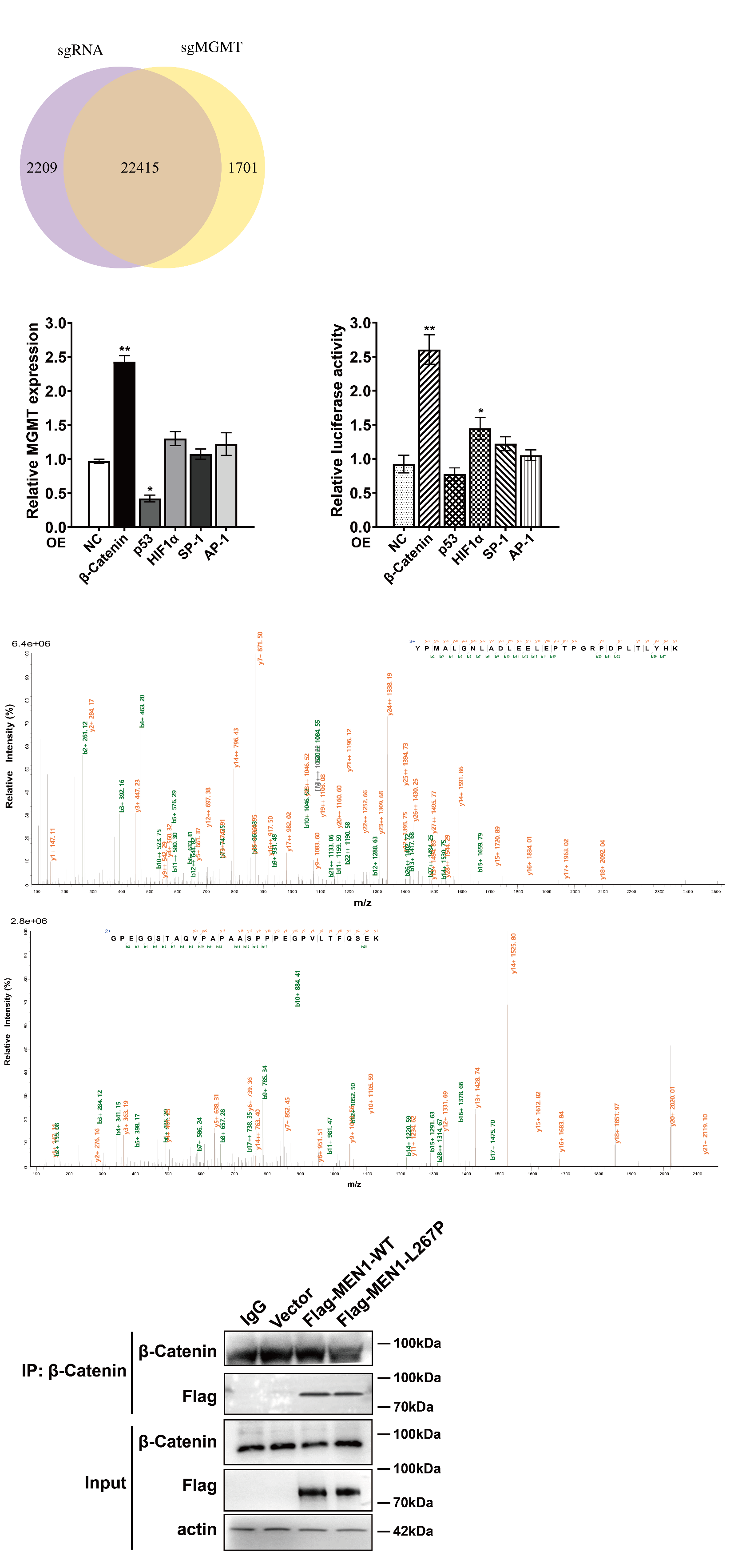


**B**

**A**

**Figure S5. A) Quantification of MGMT mRNA expression in the overexpression of reported different transcription factors.** QGP-1 cells were transfected individually by indicated transcription factor plasmids for 36hr, and subjected to RT-qPCR analysis for MGMT mRNA with actin as a control (mean ± SD, **P<0.01, *P<0.05, n = 3). **B) Determination of MGMT promoter activity in the overexpression of reported different transcription factors.** QGP-1 cells were co-transfected with the reporter MGMT-luc construct and indicated transcription factor plasmids for 36hr, and subjected to the determination luciferase activity. TK-Renilla luciferase plasmid was included in each transfection to normalize transfection efficiency (mean ± SD, **P<0.01, *P<0.05, n = 3).

**A**


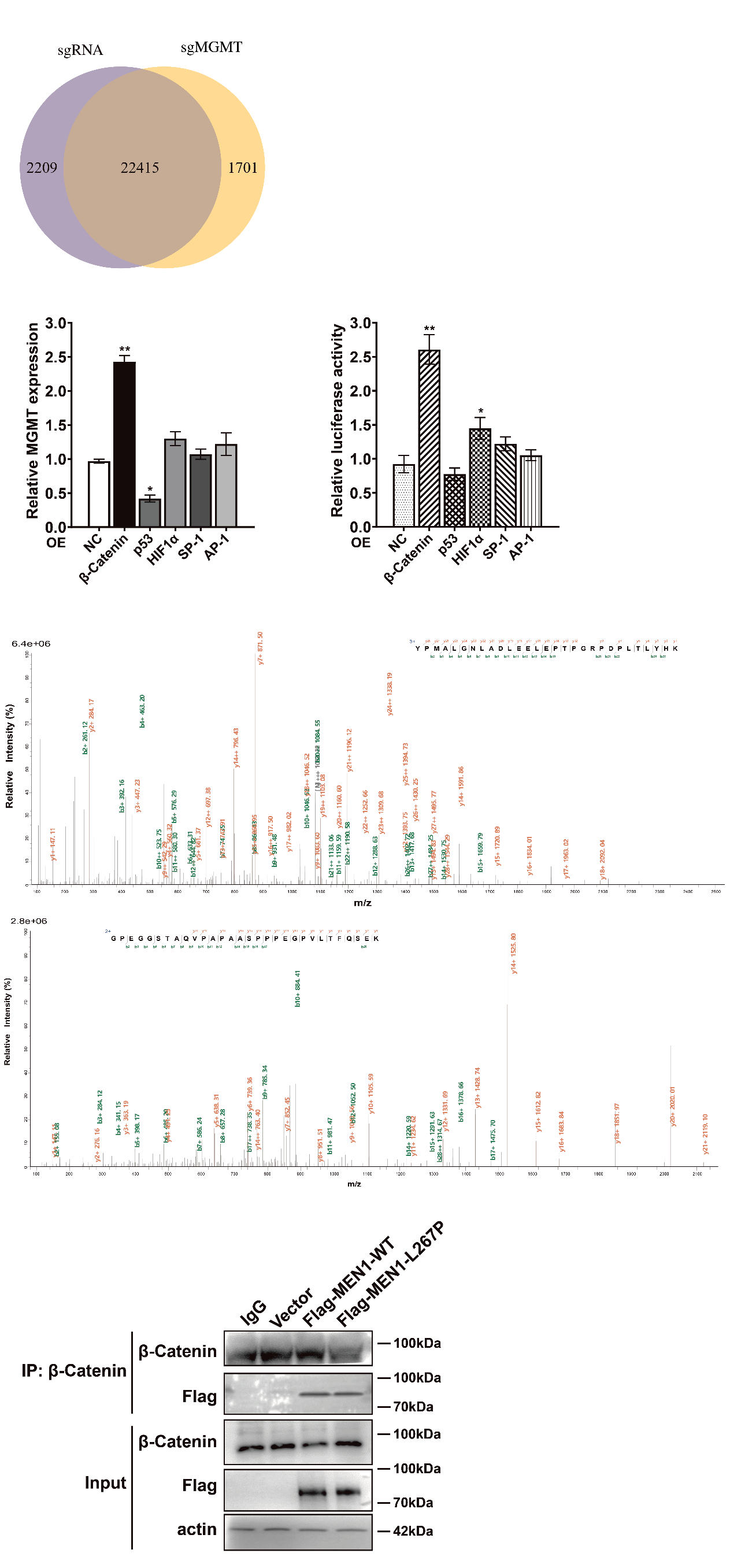


**B**


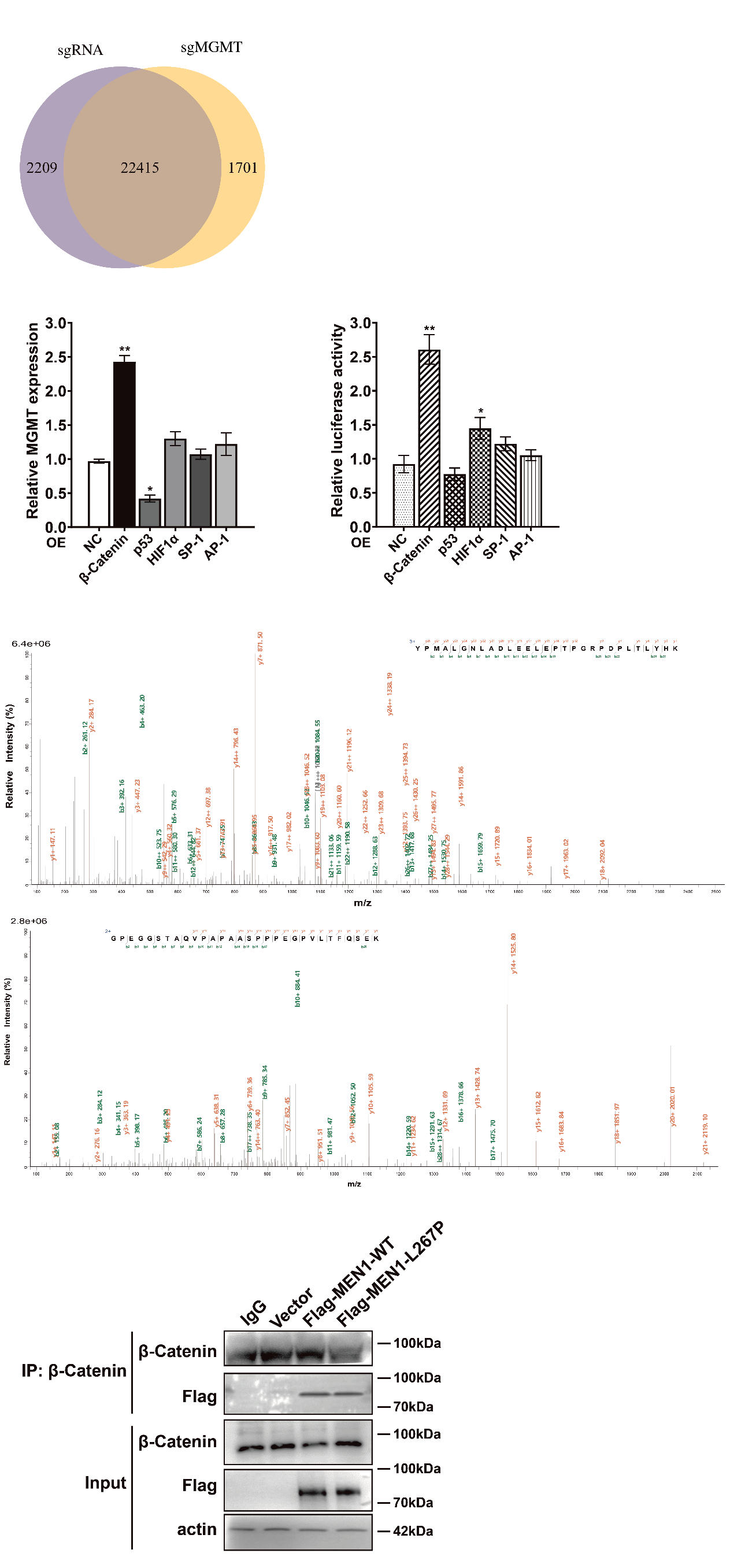


**Figure S6. A)** Representative tandem MS spectrum of the YPMALGNLADLEELEPTPGRPDPLTLYHK peptide from MEN1 as determined by IP-Mass Spec. B) Representative tandem MS spectrum of the GPEGGSTAQVPAPAASPPPEGPVLTFQSEK peptide from MEN1 as determined by IP-Mass Spec.


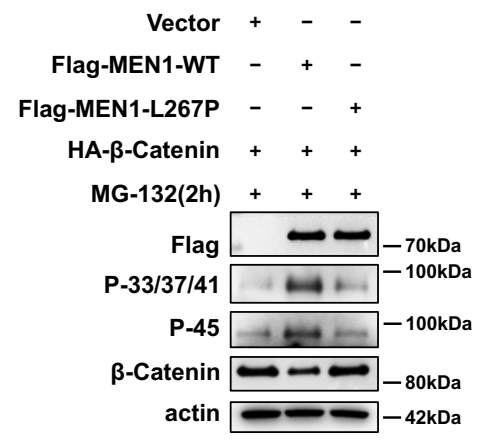


**Figure S7. The L267P of MEN1 decreased phosphor-β-catenin.** Western blot analyses of phospho-33/37/41-β-catenin (P-33/37/41) and phospho-45-β-catenin (P-45) in 293T cells transfected with HA–β-catenin and MEN1 or vector control. The transfected cells were treated with the proteasome inhibitor MG132 for 2 h before the cells were harvested. The data shown represent three independent experiments.


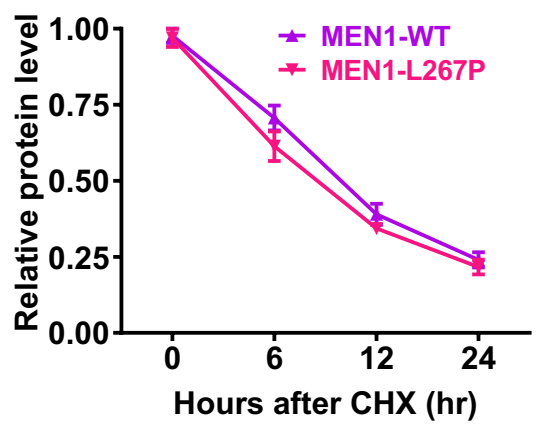

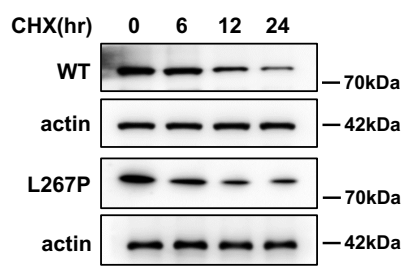


**Figure S8. No significant difference of half-life between WT and L267P of MEN1.** WT or L267P of MEN1-overexpressing stable Bon-1 cells were treated by 50 μg/mL CHX for indicated time points, and subjected to immunoblotting using antibody against Flag with actin as a loading control. The protein level was quantified by densitometry. Two-sided t test.


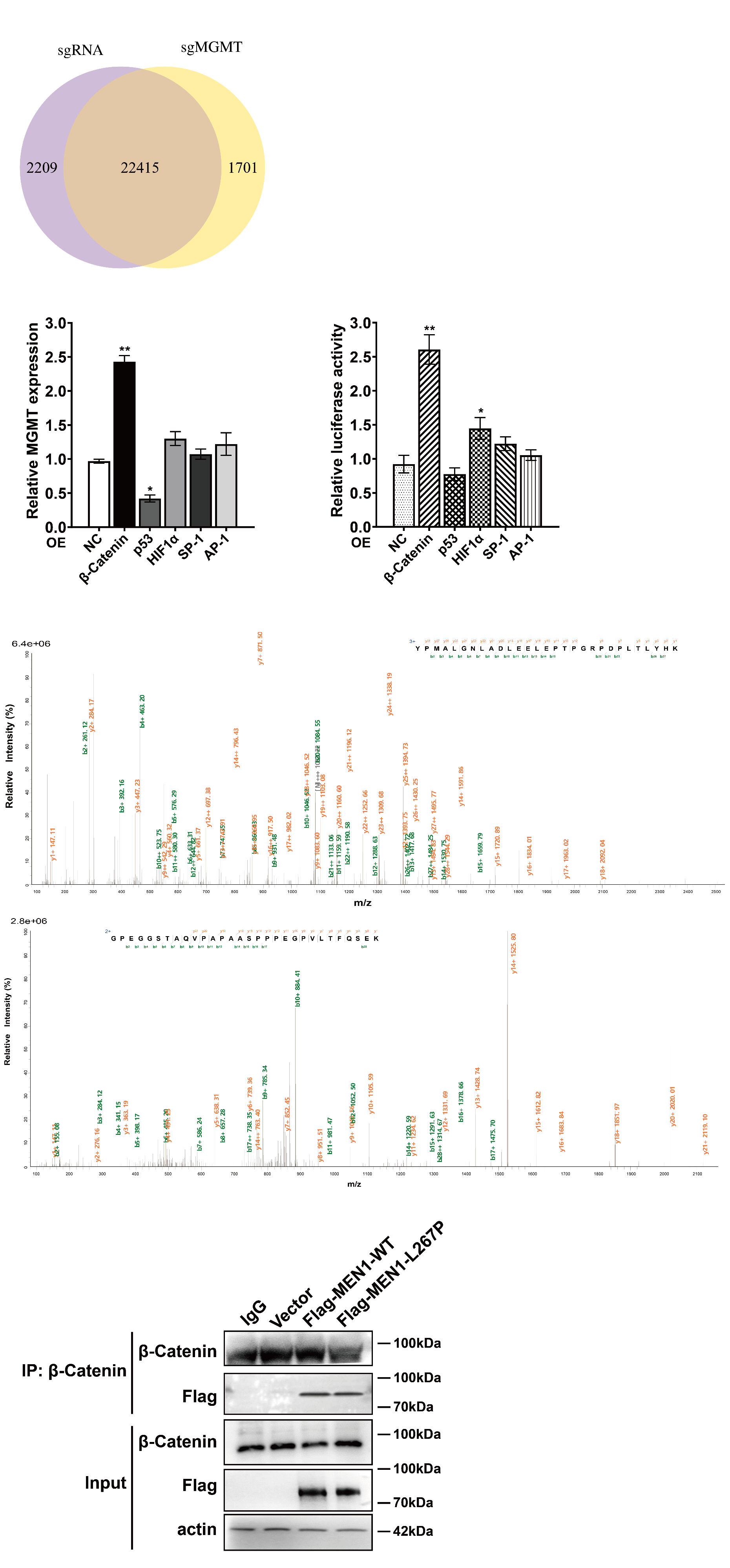


**Figure S9. The Leu267 of MEN1 was not required for interaction with β-TrCP.** MEN1-knocking out QGP-1 cells stably re-expressing indicated forms of MEN1 and negative control were subjected to immunoprecipitation using anti-β-Catenin antibody and immunoblotting using anti-Flag antibody with actin as an input control.

**Table S1** Clinicopathologic parameters of 121 PanNET patients

| **Age** | **Gender(1 male;0 female)** | **Size** | **Location（1 head;2 body & tail;3 other）** | **Grade (1 G1; 2 G2; 3 G3)** | **T (1:T1/2:T2/3:T3/4:T4)** | **N (0:N0/1:N1)** | **M (0: M0/1: M1)** | **Ki67** | **Inpatient code** | **Class** | **OS** |
| --- | --- | --- | --- | --- | --- | --- | --- | --- | --- | --- | --- |
| 48 | 0 | 10 | 2 | 2 | 4 | 1 | 0 | 4 | 352305 | T | 25.3 |
| 64 | 1 | 2 | 1 | 1 | 2 | 0 | 0 | 2 | 363336 | T | 60 |
| 59 | 0 | 2.8 | 2 | 1 | 2 | 0 | 0 | 1 | 442978 | T | 74 |
| 52 | 0 | 1.6 | 2 | 1 | 1 | 0 | 0 | 1 | 449358 | T | 65 |
| 58 | 0 | 2 | 2 | 1 | 1 | 0 | 0 | 1 | 475156 | T | 49 |
| 53 | 0 | 2.2 | 3 | 2 | 2 | 1 | 0 | 5 | 491341 | T | 42 |
| 56 | 0 | 0.8 | 1 | 1 | 1 | 0 | 0 | 1 | 491715 | T | 40 |
| 53 | 0 | 1.2 | 2 | 1 | 2 | 0 | 0 | 2 | 488685 | T | 27 |
| 44 | 0 | 2 | 2 | 2 | 1 | 0 | 0 | 10 | 495294 | T | 15.9 |
| 60 | 0 | 4 | 3 | 2 | 2 | 1 | 0 | 5 | 519360 | T | 33 |
| 70 | 0 | 3.5 | 1 | 2 | 2 | 0 | 0 | 18 | 543785 | T | 22.4 |
| 47 | 0 | 1.5 | 2 | 2 | 2 | 0 | 0 | 5 | 581332 | T | 62.7 |
| 42 | 0 | 1.5 | 1 | 1 | 1 | 0 | 0 | 1 | 575549 | T | 20.4 |
| 43 | 1 | 3.8 | 1 | 3 | 3 | 1 | 1 | 0 | 575626 | T | 45 |
| 61 | 1 | 2 | 2 | 2 | 3 | 1 | 1 | 0 | 601511 | T | 17 |
| 37 | 0 | 3 | 1 | 1 | 2 | 0 | 0 | 0 | 323132 | T | 98 |
| 53 | 0 | 1.5 | 2 | 1 | 1 | 0 | 0 | 1 | 330527 | T | 68 |
| 56 | 1 | 5 | 2 | 2 | 4 | 1 | 1 | 5 | 2007273 | T | 2 |
| 70 | 1 | 1.5 | 1 | 2 | 1 | 0 | 0 | 5 | 2008444 | T | 0.6 |
| 68 | 0 | 6 | 1 | 1 | 3 | 0 | 0 | 2 | 400649 | T | 53.5 |
| 68 | 1 | 0.5 | 1 | 1 | 1 | 0 | 0 | 1 | 493843 | T | 38 |
| 56 | 1 | 6 | 2 | 1 | 2 | 0 | 0 | 1 | 547987 | T | 16 |
| 50 | 0 | 1.8 | 2 | 2 | 1 | 0 | 0 | 10 | 2007621 | T | 24.8 |
| 63 | 1 | 0.8 | 3 | 2 | 1 | 0 | 0 | 3 | 2007930 | T | 27 |
| 73 | 1 | 6.5 | 3 | 2 | 3 | 0 | 0 | 3 | 446587 | T | 68 |
| 63 | 1 | 2 | 2 | 2 | 2 | 0 | 1 | 15 | 451745 | T | 63 |
| 61 | 1 | 3.8 | 2 | 3 | 2 | 1 | 0 | 90 | 460373 | T | 62 |
| 51 | 0 | 5.5 | 2 | 1 | 2 | 0 | 0 | 2 | 486431 | T | 28 |
| 62 | 0 | 1.2 | 2 | 1 | 1 | 0 | 0 | 2 | 522800 | T | 36 |
| 55 | 0 | 1.5 | 1 | 1 | 1 | 0 | 0 | 1 | 545585 | T | 22 |
| 65 | 1 | 2.5 | 2 | 2 | 1 | 0 | 0 | 3 | 571323 | T | 33 |
| 49 | 0 | 3.5 | 2 | 2 | 2 | 0 | 0 | 0 | 629152 | T | 31.5 |
| 57 | 0 | 3 | 1 | 1 | 2 | 0 | 0 | 1 | 639805 | T | 27.3 |
| 54 | 0 | 9.5 | 2 | 1 | 3 | 0 | 0 | 1 | 328142 | T | 98.6 |
| 75 | 1 | 6 | 1 | 2 | 4 | 1 | 1 | 5 | 336065 | T | 53 |
| 45 | 1 | 8.2 | 2 | 1 | 3 | 0 | 0 | 2 | 337517 | T | 31 |
| 42 | 1 | 5.5 | 2 | 3 | 2 | 0 | 0 | 60 | 351572 | T | 15.3 |
| 25 | 0 | 4 | 2 | 2 | 2 | 0 | 2 | 5 | 356558 | T | 46.8 |
| 52 | 1 | 5.6 | 1 | 2 | 3 | 0 | 0 | 4 | 357499 | T | 39 |
| 34 | 0 | 10.5 | 2 | 2 | 3 | 0 | 0 | 5 | 359110 | T | 25.8 |
| 50 | 0 | 1.3 | 1 | 2 | 2 | 1 | 0 | 6 | 387136 | T | 32 |
| 31 | 0 | 6 | 1 | 2 | 2 | 0 | 0 | 0 | 392426 | T | 77.4 |
| 56 | 0 | 2 | 3 | 1 | 2 | 0 | 0 | 2 | 395187 | T | 51.9 |
| 41 | 0 | 1.1 | 1 | 1 | 1 | 0 | 0 | 2 | 492648 | T | 73 |
| 62 | 0 | 9 | 2 | 2 | 4 | 1 | 1 | 8 | 533764 | T | 7.5 |
| 54 | 0 | 6.5 | 2 | 2 | 2 | 0 | 0 | 3 | 567887 | T | 15.3 |
| 63 | 1 | 1.5 | 3 | 1 | 2 | 1 | 0 | 1 | 577405 | T | 22.5 |
| 70 | 1 | 6 | 1 | 2 | 3 | 1 | 0 | 3 | 577675 | T | 20 |
| 38 | 1 | 4 | 3 | 1 | 3 | 1 | 0 | 1 | 445543 | T | 64 |
| 32 | 1 | 4 | 2 | 2 | 4 | 1 | 1 | 15 | 491382 | T | 38 |
| 69 | 0 | 2.5 | 2 | 1 | 2 | 0 | 0 | 2 | 531563 | T | 32 |
| 61 | 0 | 1.2 | 2 | 2 | 1 | 0 | 0 | 4 | 526660 | T | 20 |
| 44 | 0 | 4 | 2 | 2 | 2 | 0 | 0 | 5 | 361330 | T | 89 |
| 49 | 1 | 3.5 | 2 | 2 | 2 | 0 | 0 | 5 | 408402 | T | 74 |
| 67 | 1 | 3 | 1 | 1 | 2 | 1 | 0 | 1 | 426381 | T | 68 |
| 30 | 0 | 6 | 2 | 2 | 4 | 1 | 1 | 10 | 430716 | T | 65 |
| 39 | 0 | 5.5 | 2 | 2 | 3 | 1 | 0 | 5 | 466090 | T | 58 |
| 41 | 0 | 4.5 | 2 | 3 | 3 | 1 | 0 | 28 | 470373 | T | 55 |
| 50 | 0 | 3 | 1 | 2 | 2 | 0 | 0 | 5 | 485041 | T | 41 |
| 46 | 0 | 9 | 1 | 2 | 2 | 0 | 0 | 8 | 504916 | T | 42.7 |
| 58 | 0 | 3.5 | 2 | 3 | 2 | 0 | 0 | 60 | 480896 | T | 17 |
| 63 | 1 | 2.5 | 2 | 2 | 3 | 1 | 0 | 15 | 506646 | T | 56 |
| 53 | 0 | 2.2 | 2 | 1 | 2 | 0 | 0 | 2 | 514967 | T | 11.5 |
| 47 | 0 | 3.5 | 1 | 2 | 2 | 0 | 0 | 10 | 531236 | T | 32 |
| 49 | 0 | 1.6 | 2 | 2 | 2 | 0 | 0 | 5 | 513394 | T | 17.5 |
| 63 | 1 | 2.5 | 1 | 2 | 2 | 0 | 0 | 5 | 533184 | T | 28 |
| 55 | 0 | 3.5 | 1 | 1 | 2 | 0 | 0 | 2 | 537725 | T | 25.2 |
| 66 | 1 | 3.5 | 2 | 2 | 3 | 1 | 0 | 15 | 520880 | T | 24 |
| 60 | 0 | 5 | 1 | 2 | 2 | 0 | 0 | 10 | 329552 | T | 23.7 |
| 72 | 1 | 5 | 2 | 2 | 2 | 1 | 0 | 5 | 543366 | T | 19 |
| 28 | 1 | 3 | 1 | 2 | 2 | 0 | 0 | 4 | 545138 | T | 22 |
| 70 | 0 | 3.5 | 2 | 2 | 3 | 0 | 1 | 3 | 545636 | T | 3.3 |
| 56 | 1 | 3 | 1 | 2 | 2 | 0 | 0 | 3 | 554562 | T | 12.5 |
| 44 | 0 | 1.5 | 1 | 2 | 4 | 1 | 1 | 20 | 547111 | T | 50.5 |
| 68 | 1 | 1.5 | 2 | 3 | 1 | 0 | 0 | 40 | 560140 | T | 5.5 |
| 67 | 1 | 2.5 | 1 | 2 | 2 | 0 | 0 | 15 | 560368 | T | 25 |
| 55 | 0 | 3.5 | 1 | 2 | 3 | 1 | 0 | 5 | 560467 | T | 2 |
| 40 | 0 | 2.2 | 2 | 2 | 2 | 1 | 0 | 5 | 580865 | T | 35 |
| 59 | 0 | 1.8 | 1 | 2 | 1 | 0 | 0 | 3 | 581079 | T | 39.3 |
| 58 | 0 | 4 | 1 | 1 | 3 | 1 | 0 | 1 | 583596 | T | 99 |
| 55 | 0 | 3 | 1 | 1 | 2 | 0 | 0 | 2 | 570750 | T | 24 |
| 37 | 1 | 11 | 2 | 2 | 2 | 0 | 0 | 4 | 573095 | T | 13 |
| 52 | 0 | 2 | 1 | 1 | 2 | 0 | 0 | 1 | 578302 | T | 21 |
| 38 | 0 | 2.5 | 1 | 3 | 1 | 0 | 0 | 20 | 576898 | T | 18.3 |
| 60 | 0 | 3.2 | 2 | 2 | 3 | 1 | 0 | 5 | 616229 | T | 99 |
| 53 | 1 | 2 | 2 | 2 | 2 | 1 | 0 | 3 | 621149 | T | 23.7 |
| 39 | 0 | 1.8 | 2 | 2 | 1 | 0 | 0 | 15 | 619198 | T | 80.5 |
| 40 | 1 | 4 | 1 | 2 | 3 | 0 | 0 | 5 | 625138 | T | 66.6 |
| 44 | 1 | 3.5 | 2 | 2 | 3 | 1 | 0 | 5 | 2002346 | T | 26 |
| 41 | 1 | 2.5 | 2 | 2 | 2 | 0 | 0 | 3 | 471787 | T | 51 |
| 44 | 0 | 3 | 2 | 1 | 2 | 0 | 0 | 2 | 495795 | T | 38 |
| 34 | 1 | 2 | 2 | 1 | 3 | 0 | 0 | 2 | 542951 | T | 19 |
| 61 | 0 | 7.5 | 2 | 2 | 2 | 0 | 0 | 3 | 588979 | T | 16 |
| 46 | 0 | 1.5 | 2 | 1 | 1 | 0 | 0 | 2 | 605630 | T | 39 |
| 71 | 1 | 3.5 | 2 | 2 | 2 | 0 | 0 | 10 | 377039 | T | 13 |
| 56 | 0 | 5 | 2 | 2 | 3 | 1 | 1 | 19 | 404812 | T | 39.9 |
| 59 | 1 | 6 | 1 | 2 | 1 | 1 | 0 | 18 | 404550 | T | 49.0 |
| 44 | 0 | 14 | 2 | 2 | 2 | 0 | 0 | 3 | 407467 | T | 104 |
| 72 | 1 | 4 | 1 | 2 | 2 | 1 | 1 | 13 | 414219 | T | 97 |
| 52 | 0 | 2.5 | 2 | 2 | 2 | 0 | 0 | 10 | 429922 | T | 82 |
| 48 | 1 | 6 | 2 | 2 | 2 | 1 | 0 | 16 | 426955 | T | 8.3 |
| 55 | 1 | 4.5 | 1 | 2 | 3 | 1 | 0 | 3 | 510215 | T | 24 |
| 53 | 0 | 4.5 | 2 | 2 | 2 | 0 | 0 | 4 | 526205 | T | 33 |
| 43 | 1 | 5 | 2 | 3 | 2 | 0 | 0 | 50 | 531922 | T | 31 |
| 56 | 0 | 6.5 | 2 | 2 | 2 | 0 | 1 | 10 | 536048 | T | 25.2 |
| 51 | 1 | 4 | 1 | 2 | 2 | 0 | 0 | 5 | 536380 | T | 20.3 |
| 56 | 0 | 1 | 1 | 3 | 3 | 1 | 0 | 25 | 524364 | T | 19 |
| 64 | 1 | 2.5 | 2 | 2 | 2 | 0 | 0 | 5 | 546264 | T | 21 |
| 25 | 1 | 2.5 | 2 | 1 | 2 | 0 | 0 | 1 | 547995 | T | 53.4 |
| 56 | 1 | 4 | 1 | 2 | 2 | 1 | 1 | 5 | 552470 | T | 3.6 |
| 61 | 1 | 8 | 2 | 1 | 3 | 0 | 0 | 2 | 581002 | T | 35 |
| 65 | 0 | 1.5 | 1 | 1 | 2 | 0 | 0 | 1 | 581958 | T | 62 |
| 52 | 1 | 5.5 | 2 | 2 | 3 | 1 | 1 | 5 | 584155 | T | 49 |
| 69 | 1 | 5 | 2 | 2 | 3 | 0 | 0 | 5 | 571733 | T | 41 |
| 80 | 0 | 5.5 | 2 | 2 | 2 | 0 | 0 | 5 | 627931 | T | 1 |
| 76 | 0 | 4.5 | 2 | 2 | 2 | 0 | 0 | 10 | 628473 | T | 13 |
| 48 | 1 | 10.5 | 2 | 1 | 2 | 0 | 0 | 4 | 612571 | T | 7 |
| 48 | 1 | 5.5 | 2 | 2 | 2 | 0 | 0 | 8 | 648104 | T | 27 |
| 73 | 1 | 9.5 | 2 | 1 | 4 | 1 | 1 | 2 | 424054 | T | 34.4 |
| 46 | 0 | 9 | 2 | 3 | 4 | 1 | 1 | 25 | 526760 | T | 14 |
| 77 | 1 | 4 | 1 | 2 | 4 | 0 | 0 | 5 | 473704 | T | 51 |

| Characteristics | Overall | MGMT | | P-value |
| --- | --- | --- | --- | --- |
|  | No. (%) | Low | High |  |
| **Age** |  |  |  | 0.317 |
| ＜55 | 60(49.6) | 27(22.3) | 33(27.3) |  |
| ≥55 | 61(50.4) | 33(27.3) | 28(23.1) |  |
| **Gender** |  |  |  | 0.529 |
| Female | 68(56.2) | 32(26.4) | 36(29.8) |  |
| Male | 53(43.8) | 28(23.1) | 25(20.7) |  |
| **Tumor size(cm)** |  |  |  | 0.609 |
| ≤4cm | 80(66.1) | 41(33.9) | 39(32.2) |  |
| ＞4cm | 41(33.9) | 19(15.7) | 22(18.2) |  |
| **Location** |  |  |  | 0.209 |
| Head | 43(35.5) | 17(14.0) | 26(21.5) |  |
| Body & tail | 71(58.7) | 38(31.4) | 33(27.3) |  |
| Other | 7(5.8) | 5(4.1) | 2(1.7) |  |
| **AJCC 8th TNM stage** |  |  |  | 0.655 |
| I | 21(17.4) | 8(6.7) | 13(10.7) |  |
| II | 63(52.1) | 33(27.3) | 30(24.8) |  |
| III | 27(22.3) | 13(10.7) | 14(11.6) |  |
| IV | 10(8.2) | 6(5.0) | 4(3.3) |  |
| **Node status** |  |  |  | 0.741 |
| N0 | 83(68.6) | 42(34.7) | 41(33.9) |  |
| N1 | 38(31.4) | 18(14.9) | 20(16.5) |  |
| **Metastasis** |  |  |  | 0.583 |
| M0 | 103(85.1) | 50(41.3) | 53(43.8) |  |
| M1 | 18(14.9) | 10(8.3) | 8(6.6) |  |
| **WHO classification** |  |  |  | 0.536 |
| G1 | 37(30.6) | 21(17.4) | 16(13.2) |  |
| G2 | 74(61.2) | 35(28.9) | 39(32.3) |  |
| G3 | 10(8.2) | 4(3.3) | 6(5.0) |  |

**Table S2.** MGMT expression stratified by selected clinicopathologic parameters of PanNET patients

Table S3. Association of MGMT expression with PFS in PanNET patients

|  |  | Univariate analysis | 2-sided | Multivariable-adjusted* | 2-sided |
| --- | --- | --- | --- | --- | --- |
| Factors | n (%) | HR (95% CI) | *P*-value | HR (95% CI) | *P*-value |
| **MGMT** |  |  |  |  |  |
| Low | 60(49.6) | 1.00(Reference) |  | 1.00(Reference) |  |
| High | 61(50.4) | 1.95(1.14-3.33) | 0.015 | 1.88(1.07-3.31) | 0.027 |

*Multivariable-adjusted Cox proportional hazards model, adjusted for age, gender, tumor size, tumor location, grade and T/N/M stage.

HR: hazard ratio; CI: confidence interval.
